# Using financial incentives to promote physical activity in American Indian adolescents: A randomized controlled trial

**DOI:** 10.1371/journal.pone.0198390

**Published:** 2018-06-01

**Authors:** Kevin R. Short, Jennifer Q. Chadwick, Tamela K. Cannady, Dannielle E. Branam, David F. Wharton, Mary A. Tullier, David M. Thompson, Kenneth C. Copeland

**Affiliations:** 1 Department of Pediatrics, University of Oklahoma Health Sciences Center, Oklahoma City, OK, United States of America; 2 Choctaw Nation of Oklahoma, Durant, OK, United States of America; 3 Department of Biostatistics and Epidemiology, University of Oklahoma Health Sciences Center, Oklahoma City, OK, United States of America; University of Tennessee Health Science Center, UNITED STATES

## Abstract

American Indians (AI) have high prevalence of diabetes in youth and may benefit from increasing physical activity as a strategy to improve metabolic health. We tested whether financial incentives would elicit greater frequency and/or duration of exercise in AI youth at high risk for developing diabetes. Overweight/obese AI boys and girls, 11–20 years old, were instructed to exercise on 3 days/week for 48 weeks at a tribal wellness center. The program was divided into three, 16-week-long phases to test different financial incentive strategies. Within each phase participants were randomly assigned to one of two groups that received different payments for exercise. Phase 1 was designed to test whether the size of the incentive would affect exercise frequency. In Phase 1, the number of exercise sessions did not differ between the group receiving a modest fixed-value payment per exercise session and the group receiving enhanced incentives to exercise more frequently (26 ± 3 versus 28 ± 2 sessions, respectively, p = 0.568). In Phase 2, the provision of an enhanced financial incentive to increase exercise duration resulted longer sessions, as the incentivized and standard payment groups exercised 38 ± 2 versus 29 ± 1 minutes per session (p = 0.002), respectively. In Phase 3, the effect of reducing the incentives on maintenance of exercise behaviors was inconclusive due to high participant withdrawal. Aerobic fitness increased 10% during Phase 1 but was unchanged thereafter. Insulin sensitivity and body composition were unchanged during the study. In conclusion, enhanced financial incentives increased the duration of exercise sessions, but had minimal effects on exercise participation. These results indicate that financial incentives hold promise in motivating previously sedentary, overweight/obese adolescents to exercise longer, but motivating them to sustain an exercise program remains the major challenge.

**Trial Registration**: ClinicalTrials.gov NCT01848353.

## Introduction

The metabolic and cardiovascular health of many children in the United States is poor. Thirty percent of children in the United States are overweight or obese and fewer than half are aerobically fit or reach the recommended level of daily physical activity [1–5]. Cardiorespiratory fitness in late adolescence is a significant predictor for future cardiovascular events during adulthood [6]. Several lines of evidence show that obesity and sedentary lifestyle elevate cardiometabolic risk early in life and set the stage for future disease in adulthood [7]. Thus, there is a need to develop effective interventions to improve physical fitness and metabolic health in children and adolescents, especially those who are obese.

The need to prevent cardiometabolic diseases, particularly type 2 diabetes (T2D), is especially important for American Indian youth. American Indian adolescents have much higher rates of overweight (53.5%) or obesity (33.8%) than national averages [8]. They also have higher prevalence rates of impaired fasting glucose and insulin resistance than African American and non-Hispanic white adolescents [9]. American Indian adolescents have the highest incidence of T2D compared to other racial and ethnic groups in the US and these incidence rates have increased over the past 15 years [10].

Physical activity is an effective strategy to improve aerobic fitness and reduce insulin resistance in overweight/obese children [11, 12]. A significant challenge, however, is modifying behavior of habitually sedentary adolescents to increase physical activity beyond a few weeks of an intervention [13]. In this study, we used a behavioral economics approach to test whether financial incentives would effectively promote exercise behavior in overweight/obese American Indian adolescents. The clinical and functional benefits of an exercise program may take several weeks to become apparent, but short-term temptations to deviate away from target behaviors may over-ride long-term goals [14]. A financial incentive can potentially help overcome this problem because the provision of money provides a tangible, nearly immediate reinforcement of target behavior and can offset the temptation of short-term choices that are contrary to long-term benefits [15]. This could be particularly important for adolescents, who tend to make decisions more on present conditions than on longer-term outcomes [16].

Prior studies with adults showed that financial payments for physical activity can promote an increase in daily walking [17, 18] or exercise participation at a fitness center [19, 20] for up to 12 months. Financial incentives were also effective at promoting use of an on-campus fitness center by college students [21, 22]. To our knowledge, only one study has used financial incentives to promote physical activity in children [23]. In a randomized controlled trial, Finkelstein and colleagues [23], used pedometers to record daily steps performed by Singaporean children, 6–12 years old, for 6–10 months. Children who could earn gift cards for meeting daily walking goals recorded higher physical activity than an unpaid control group. That investigation, and those performed with college students [21, 22], demonstrate that financial incentives may have value for promoting physical activity in adolescents and young adults [21–23]. However, those prior studies included participants with an average body mass index (BMI) within the normal range, who were generally healthy, and had a wide initial range in habitual physical activity. Thus, it is unclear whether a similar financial incentive strategy would be effective for promoting exercise behavior in other populations of adolescents, especially those who are overweight or obese, and habitually sedentary.

The primary objective of the current investigation was to test the hypothesis that financial incentives would encourage overweight/obese American Indian youth with habitually low physical activity to establish and maintain better exercise habits. The provision of financial incentives was designed to reinforce the frequency and duration of exercise at tribal wellness centers that were recently built in several small-town communities in Southeast Oklahoma. The study was also designed to test the durability of exercise behavior when financial rewards were reduced or withdrawn.

## Materials and methods

### Design

This was a prospective, randomized trial, with exercise behavior (frequency and duration of exercise) as the primary outcome, and insulin sensitivity, aerobic fitness, and body composition as secondary outcomes. The study was approved by the Institutional Review Boards (IRB) of the Choctaw Nation of Oklahoma (Protocol Number 12–0162), and the University of Oklahoma Health Sciences Center (OUHSC, Protocol Number 0434), respectively. The trial was registered at ClinicalTrials.gov (NCT01848353) on April 25, 2013. Participants were enrolled for the exercise intervention from July 2013—April 2016. During preliminary testing, the protocol was modified to replace the planned oral glucose tolerance tests with a measurement of insulin sensitivity that required only fasting blood collection. This change was made to reduce the time demands for participants and staff. In March 2015, we revised the age and body mass index (BMI) eligibility criteria for the exercise intervention, as described in the Participants section below. This change was made to increase the rate of enrollment, although other recruitment strategies, such as increased advertising in the tribal newspaper and presentations a community events were ultimately more successful. At that time, we also added a group of normal weight peers from the same communities, to serve as a reference group in baseline comparisons. Since that reference group did not participate in the incentivized exercise intervention, their results are not included in the current report. All changes to the protocol and study procedures were approved by both IRBs. The protocol and Consolidated Standards of Reporting Trials (CONSORT) checklist are available as supporting documents S1 Checklist and S1 Protocol, respectively.

The exercise intervention was 48-weeks in duration and subdivided into three, consecutive 16-week phases. Each phase was designed to test how different incentive schemes would affect exercise frequency and/or duration. Before each study phase, all eligible participants were randomized to one of two groups that received different payments for their exercise behavior, as described below.

### Participants

Male and female American Indians were recruited from the Choctaw Nation Health Service Area of Southeast Oklahoma. From July 2013 through March 2015 the age criterion for the exercise intervention was 11,0 to 17.9 years old, and the BMI criterion was ≥ 95^th^ percentile for age- and sex-specific norms based on growth charts from the Centers for Disease Control and Prevention [24], respectively. In April 2015, the age criterion was changed to 11.0 to 20.9 years old and the BMI criterion was changed ≥ 85^th^ percentile. Those changes were made to increase enrollment and the generalizability of the results, without compromising the study objectives. The other primary eligibility criteria throughout the study were: American Indian, as certified by tribal enrollment, sexual maturation level determined by a pediatrician as ≥ Tanner stage 2 for breasts (girls) or genitalia (boys) [25, 26], self-reported history of T2D in a first or second degree relative, and low physical activity during the three months prior to enrollment. Low physical activity was defined as attaining (via self-report) less than 30 minutes of structured moderate-to-vigorous intensity exercise on three or fewer days/week over the preceding three months. Participants were excluded if they had confirmed diabetes or other potentially confounding metabolic disorders, were unable to safely exercise as determined by a physician, or used medications known to influence the stated outcomes. Preliminary eligibility was assessed during an initial phone call or in-person discussion with the study participant and/or their parent or guardian. Participants and their parents or guardians provided their informed, written consent and assent at the first visit in accordance with IRB guidelines. After enrollment, a qualified medical provider conducted a medical exam to assure eligibility for the study.

### Exercise training and monitoring

Participants completed exercise sessions on schedules of their own choosing, at one of five wellness centers operated by Choctaw Nation in Southeast Oklahoma. Most participants completed their exercise sessions at either Hugo or Talihina, OK. All participants were eligible to use the wellness centers at no cost. Staff at each center provided initial instruction and supervision to assure that participants completed exercise sessions safely and effectively. The design of the study permitted participants to choose any type of individual or group exercise. This could include, but was not limited to walking or running on a treadmill, indoor walkway or outdoor path, stationary cycling, stair climbing machine, elliptical machine, stickball (a traditional American Indian sport), aerobic dance (Zumba), martial arts, resistance training, or basketball. Participants could perform different activities within and between each visit to the fitness center. Our intent was for participants to discover the activities that they found most enjoyable since that has been shown to be a potential motivator for adolescents to exercise [27]. Throughout the study, participants were encouraged to complete at least three exercise sessions per week, with a minimum of 20 minutes per session of moderate-to-vigorous physical activity (MVPA). This goal is modest, as the current recommendation for children and adolescents is to perform 60 minutes of MVPA per day on at least five days per week [28]. All participants who completed at least 16 exercise sessions in Phase 1 were eligible to continue on to Phase 2. Participants who completed fewer than 16 exercise sessions in Phase 1 were involuntarily withdrawn from further study participation. The same criterion was used for the transition from Phase 2 to Phase 3. We explained to participants this involuntary withdrawal criterion, which amounted to an average of one session per week. We chose the criterion because participants could readily understand it, and because such infrequent participation was unlikely to result in any benefit.

During exercise, participants wore a personally assigned chest strap heart rate (HR) monitor (Spirit System, Interactive Health Technologies, Austin, TX). The monitor and accompanying software recorded the duration and intensity of each exercise session, calculated the time spent in MVPA, and provided feedback during and after exercise. Peak HR for each participant was measured during an aerobic fitness test, described below, and was used to calculate the HR thresholds for moderate (70% of peak HR) and vigorous (85% of peak HR) intensity. Those HR values were programed into the monitoring system so that every two minutes the monitors produced an audio signal (a series of beeps) that indicated whether the HR was in the low, moderate, or vigorous intensity range. A computer logging system provided participants visual feedback about their progress during and after each session, and stored the results in a password-protected database. To earn a payment, the minimum requirement of every exercise session was to accumulate at least 20 minutes of MVPA. Rest breaks during the sessions were allowed, but multiple exercise sessions within a day were counted as a single session for that day. Participants kept a written logbook in which they recorded the types of activities they performed and MVPA time for each session. The fitness center staff checked and signed participants’ logbooks after every exercise session. The logbook was used to confirm exercise sessions in the event of discrepancy or technical problem.

### Groups and assignment

At the beginning of each study phase, participants were randomly assigned to one of two groups. Participants were assigned in blocks of two, with both persons of the same sex and age, following a stratified blocking design developed at the start of the trial by the study statistician. An exception to the randomization was made when siblings enrolled; in that case both siblings were assigned to the same group to avoid contamination of treatment effects [23]. Because the study coordinators and wellness center staff interacted closely with participants, and were responsible for confirming the accuracy and receipt of payments to the participants, they were not blinded to group assignments.

### Group-specific incentives and payments

All participants received payments for completing exercise sessions but the size of the payments differed by group assignment. In Phase 1, the Standard Payment (Standard) group received a fixed payment of $4 for each of three qualifying exercise sessions per week and could therefore earn up to $12 per week. The Enhanced Incentive (Incentive) group received $4, $7, and $16, respectively, for the first, second, and third qualifying exercise session of the week and could therefore earn up to $30 per week. We tested the hypothesis that the frequency of exercise sessions would be higher in the Enhanced Incentive group.

In Phase 2, participants randomized to the Standard Payment (Standard) group received $4 for each of three qualifying exercise sessions that had at least 20 minutes of MVPA, which allowed them to earn up to $12 per week. The Enhanced Incentive (Incentive) group received $4 for exercise sessions with 20–39 minutes of MVPA, $7 for sessions with 40–59 minutes of MVPA, and $10 for sessions with ≥ 60 minutes of MVPA, respectively, which allowed them to earn up to $30 per week. We tested the hypothesis that exercise duration (time spent in MVPA per session) would be longer in the group whose participants who received the larger payment.

Phase 3 was designed to determine if exercise behavior developed during the preceding 32 weeks would continue when payments for exercise were diminished and/or removed. This phase of the study was exploratory. Participants in Phase 3 were randomly re-assigned to either a Ramp-down Payment (Ramp-down) or Raffle group. The maximum payments for the Ramp-down group began at $20 per week, through a payment structure that incentivized exercise time, with a bonus for completing 3 sessions in a week (S1 Table). The weekly payments were decreased over 8 weeks, reaching $0 for weeks 9–16. The Raffle group had a discontinuous reinforcement plan (S1 Table). They earned chances to win financial awards based on the number and duration of their exercise sessions each week. Random drawings were conducted to determine winners, with prizes of $10-$50 available.

Participants also earned $50 for completing a set of standardized clinical and physiological assessments at baseline (before starting the exercise program), and again at the end of each study phase. Every two weeks, participants were paid using a reloadable debit card, and received a detailed printed summary of their exercise sessions and payments. They were required to confirm the accuracy of the record and receipt of payments. So that participants were not penalized for illness or travel that prevented them from attending exercise sessions, they could perform make-up sessions within the three weeks before or following the occurrence, with study coordinator’s approval.

In addition to cash payments, newly enrolled participants were given a sport shirt with a custom logo designed for the study. After completing four exercise sessions, and thereby demonstrating their initial commitment to the program, the participants received a water bottle, a sling backpack, and three pairs of athletic socks. They also received $150 in credit to purchase athletic shoes and clothes of their choice through an on-line retailer. These non-financial incentives were selected at the recommendation of the Choctaw Nation wellness and healthcare leaders. The rationale for providing these items was to assure that the participants had appropriate shoes, clothes, and accessories to aid their comfort and confidence during their exercise sessions.

### Clinical and physiological tests

The following tests and measurements were performed at baseline (before starting the exercise program), and again at the end of each of the three study phases.

#### Anthropometry and body composition

Trained clinical staff measured Height, body mass, and waist circumference. BMI was calculated using body mass and height (kg/m^2^) and BMI percentile was calculated using growth charts from the Centers for Disease Control and Prevention [24]. Total body and regional fat and lean tissue were measured using bioelectrical impedance (Model BC-418, Tanita Corporation, Arlington Heights, IL).

#### Aerobic fitness

A bicycle ergometer test with increasing workloads was used to measure peak work output, peak rate of oxygen consumption (VO_2_peak), and heart rate. Continuous measurements of expired gases were performed with a facemask and metabolic measurement system (TrueOne 2400; ParvoMedics, Sandy, Utah) and heart rate was measured with an integrated chest-strap monitor (Polar Electro USA, Lake Success, New York).

#### Physical activity assessment

Free-living daily ambulatory activity was measured with accelerometers worn on the waist (Fitbit Zip, Fitbit Inc., San Francisco, CA) throughout the day, recording data each minute for seven days.

#### Blood analysis

Venous blood samples were collected in the morning following an overnight fast. Glycated hemoglobin (HbA1c) was measured on whole blood at the time of collection using a Siemens DCA Vantage analyzer (Tarrytown, NY). After centrifugation, aliquots of plasma and serum were stored at -80°C until analysis. Plasma glucose was measured by the glucose oxidase method (2300STAT Plus, Yellow Springs Instruments, Yellow Springs, OH). Serum insulin was measured using a chemiluminescent enzyme-linked immunosorbent assay (ELISA) from ALPCO (#80-INSHU-CH10, Salem, NH). Insulin sensitivity was calculated using glucose and insulin concentrations with the revised integrated homeostatic model of assessment (iHOMA2) [29].

#### Questionnaires

Participants completed questionnaires at baseline to assess personal and environmental concerns that could potentially interfere with their ability to complete the study. The Choctaw Nation of Oklahoma offers several social service programs designed to reduce distress, including organized activities, nutrition classes, food support, and counseling. Study staff assisted participants to access these services if they acknowledged a need, although the use of specific services was not tracked or used as part of the study outcomes. The goal of this effort was to ensure that a decision by the child or family to discontinue the study was due to their free choice, and not due to barriers that could be reasonably addressed with available services. Separate questionnaires and telephone interviews were administered after participants withdrew from the study in an informal attempt to identify perceived barriers.

### Sample size estimates

We planned the study to afford sufficient power to address the hypothesis connected with Phase 1. Because the number of completed exercise sessions likely follows a Poisson distribution, we used PASS software (v.2005, NCSS, LLC) to explore a Poisson regression model. The model verified that two groups with 40 participants in each would afford 80% power to detect between-group ratios in the number of completed exercise sessions of 1.1 to 1.4, depending on the groups’ mean exercise attendance. In anticipation of the possibility that some participants would either withdraw voluntarily or would not meet entry criteria after initial enrollment, we planned for enrollment to continue until approximately 80 participants started the exercise program in Phase 1.

Since no prior data were available to estimate rates of participation in an incentivized exercise training program for this population, we made assumptions based on our collective experience with exercise and clinical trials. We anticipated dropout rates of about 32% during Phase 1, and about 20% in each of Phases 2 and 3. We reasoned that participants who were the least motivated or who experienced the highest barriers to participation were most likely to discontinue during Phase 1 and those who remained would be more likely to complete the entire 48-week study. With respect to the hypothesis explored in Phase 2, we assumed that total exercise time during the phase would be variable (estimated standard deviation = 8 hours), so that two groups with 32 participants in each would afford 80% power to detect a between-group difference of 5.75 hours (out of the total of 48 hours possible) in mean exercise time. The hypotheses underlying all sample size estimates were two-sided and assumed a Type 1 (alpha) probability of 0.05.

### Data analyses

Since group assignments were balanced for age and sex, the groups were expected to be similar at baseline with respect to those and most other demographic variables. Comparisons were performed to identify demographic or other variables for which between-group differences might affect the primary outcomes. Subsequent analyses were planned to adjust for these potentially confounding variables. Multivariable regression models were used to assess between-group differences in the primary outcomes, and to analyze the effects of exercise training on secondary outcomes. Residuals generated from regression models were inspected for linearity, normality, and equality of variance and the data were transformed as appropriate. Between-group differences were evaluated with 95% confidence intervals. In secondary analyses, we calculated bivariable correlations to determine strength of association among selected variables, using Pearson's or Spearman's correlation coefficients, as appropriate. T-tests and Chi-square tests were used for selected between-and within-group comparisons. P values less than an alpha of 0.05 were considered significant for all tests. Statistical analyses were performed using SPSS software, version 19 (IBM, Armonk, New York).

## Results

### Phase 1

**Fig 1:**
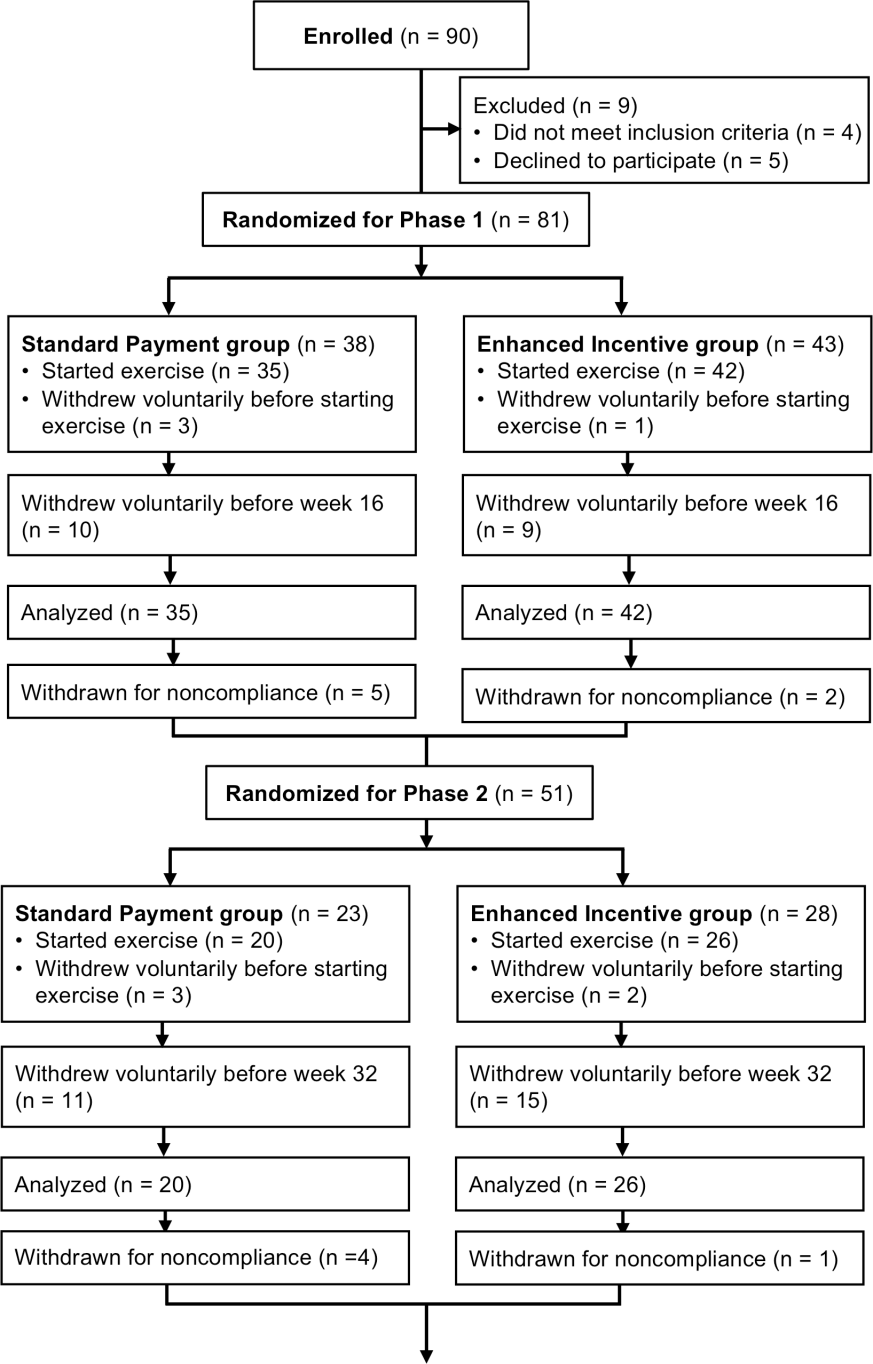
Flow chart showing the number of participants completing Phases 1 and 2. The Standard group in Phases 1 and 2 received a fixed payment for exercise sessions, while the Incentive group received enhanced payments that were meant to promote either higher exercise frequency (Phase 1) or longer exercise duration (Phase 2).

Ninety participants enrolled in the study and 81 completed the initial screening and baseline testing, at which point the randomized group assignment for Phase 1 was performed (Fig 1). Three girls and one boy voluntarily withdrew before beginning the exercise program; their data were not included in the analyses. For the 77 remaining participants assigned to either the Standard or Incentive groups, there were no between-group differences in clinical and physiological characteristics at baseline (Table 1). Twelve girls and seven boys (25% of the cohort) withdrew voluntarily before the end of Phase 1, but their exercise behavior was available for analysis. The characteristics of the participants who voluntarily withdrew during Phase 1 (Age: 15.4 ± 2.7 y; BMI: 36.6 ± 9.1 kg/m^2^, 98 ± 3 percentile) were not different from those of participants who completed Phase 1 (Age: 14.0 ± 2.2y; BMI: 34.2 ± 6.5 kg/m^2^, 98 ± 3 percentile). Participants who withdrew from Phase 1 performed fewer exercise sessions (15 ± 14, range = 1 to 47 sessions) than those who completed Phase 1 (31 ± 14, range = 7–71 sessions, p < 0.001 for comparison between completers and non-completers).

**Table 1:** Clinical and physiological characteristics at baseline, changes at the end of Phase 1, and exercise performance in Phase 1.

	Standard Group		Incentive Group	
	Baseline	Change at End of Phase	Baseline	Change at End of Phase
BMI, kg/m^2^ *	34.9 ± 8.5	0.7 ± 1.1†	34.7 ± 6.2	0.7 ± 1.8†
BMI, percentile	98 ± 3	0 ± 1	98 ± 3	0 ± 1
Body fat, %	43.6 ± 8.1	0.9 ± 2.7	43.1 ± 6.9	0.1 ± 4.2
Fat-free mass, kg	48.6 ± 10.2	0.6 ± 2.9	52.2 ± 10.2	0.9 ± 4.3
Waist circumference, cm	104 ± 16	-1 ± 5	108 ± 12	0 ± 8
VO_2_peak, ml/kg FFM/min*	34.2 ± 7.1	3.4 ± 5.7†	35.2 ± 8.7	3.7 ± 7.5†
Steps per day	6,404 ± 3,425	-869 ± 2,318	6,218 ± 2,419	483 ± 3,401
Glucose, mmol/l	5.3 ± 0.5	0.2 ± 1.0	5.1 ± 0.4	0.4 ± 2.1
Insulin, pmol/l	164.7 ± 229.3	-2.9 ± 166.9	119.7 ± 94.3	-11.4 ± 80.3
iHOMA2 (%S)	74.9 ± 48.5	-13.8 ± 40.7	68.6 ± 44.2	-4.9 ± 26.2
HbA1c (%)	5.4 ± 0.3	0.1 ± 0.2†	5.3 ± 0.2	0.1 ± 0.6
Exercise sessions performed	—	26 ± 16	—	28 ± 15
Total MVPA time, h	—	15.2 ± 10.1	—	15.0 ± 8.1
MVPA time per exercise session, minutes	—	35 ± 7	—	32 ± 8
MVPA time in moderate intensity range, %	—	63 ± 18	—	64 ± 18
Payments for exercise, USD$	—	99 ± 58	—	245 ± 144§

Values shown as mean ± SD. BMI, body mass index; VO_2_peak, peak oxygen uptake during aerobic fitness test; FFM, fat-free mass; iHOMA2, integrated homeostatic model of assessment; HbA1c, hemoglobin A1c; MVPA, moderate-to-vigorous activity. The Standard group had 18 girls, 17 boys; mean age 14.2 ± 2.4 years at baseline. The Incentive group had 25 girls, 17 boys; mean age 14.4 ± 2.3 years at baseline.

*Main effect of time for entire cohort, p < 0.01.

† Increase from baseline to end of Phase 1 within group, p < 0.03.

§ Difference between groups, p < 0.01.

Contrary to the hypothesized outcome for Phase 1, participants who received larger financial incentives did not complete more exercise sessions with at least 20 minutes of MVPA (Table 1, 95% CI for between group difference = -5.1 to 9.2 sessions). There was a broad range of exercise sessions per participant (1–71 sessions). Seven members of the Standard group and 14 members of the Incentive group completed at least 40 of the 48 possible exercise sessions, an average of 2.5 sessions per week (p = 0.191 for between group difference). An unexpectedly high number of sessions (99, or 4.6% of all sessions in Phase 1) had less than 20 minutes of MVPA and so did not qualify for payment. The number of short-duration sessions did not differ between groups.

**Fig 2:**
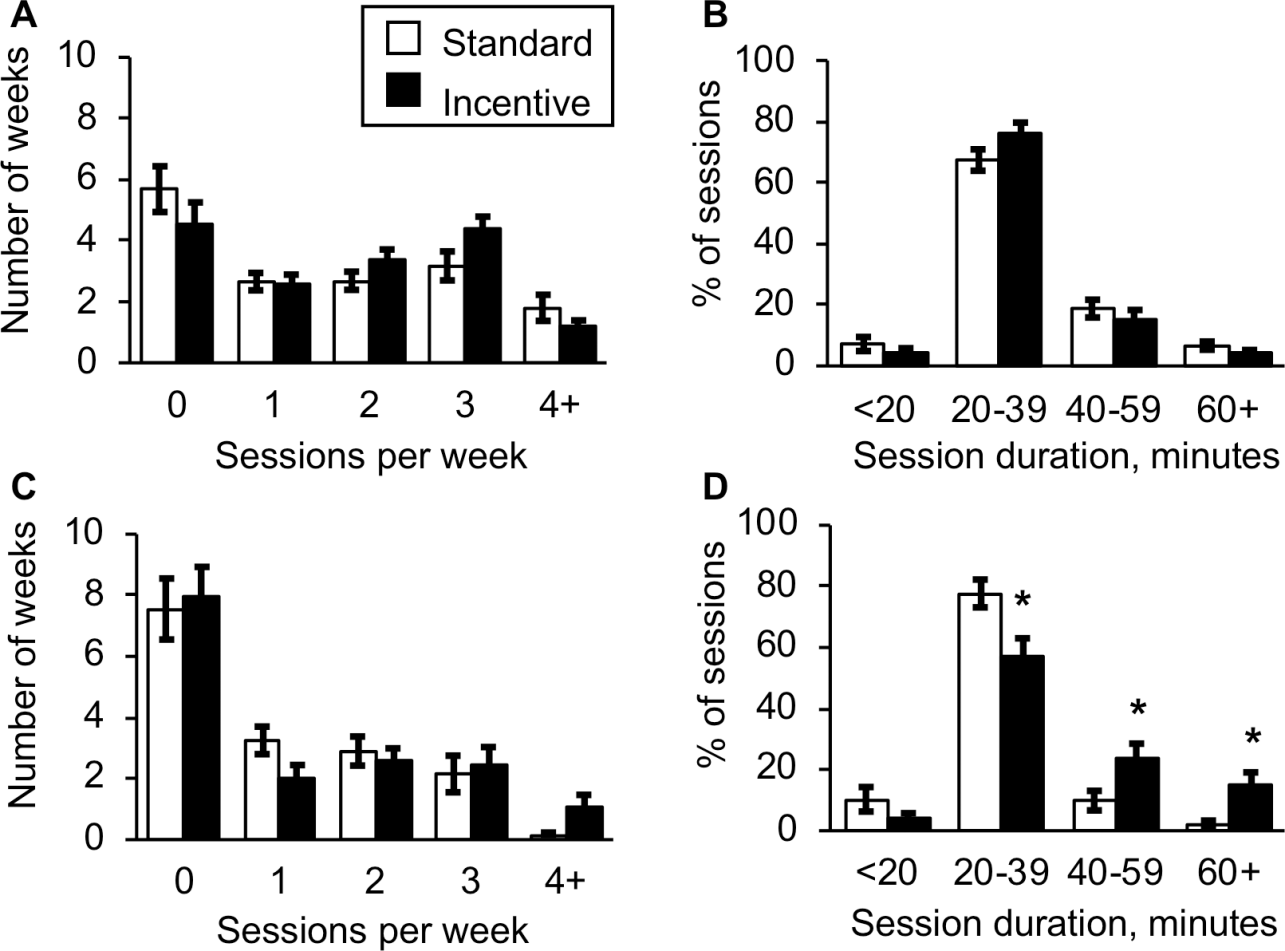
Exercise behavior in Phases 1 and 2. In Phase 1 (A & B), there were no differences between the Standard and Incentive payment groups in the distribution of sessions per week or duration per exercise session. In Phase 2 (C & D), the distribution of sessions per week did not differ between groups, but participants in the Incentive group performed more sessions that lasted at least 40 or 60 minutes, and fewer sessions in 20–39 minute range, than did those in the Standard payment group. * p < 0.03 between groups.

As a secondary analysis, we examined the frequency of weeks with zero to four-or-more exercise sessions (Fig 2A). There were no significant differences between groups for those comparisons, though the Incentive group tended to have more weeks with three sessions than the Standard group [between group difference and 95% CI: 1.2 (-0.1 to 2.5 weeks); p = 0.067]. The average MVPA time per session (Table 1), cumulative MVPA for Phase 1, and percentage of MVPA time spent in the moderate intensity range did not differ between groups. Most exercise sessions had 20–39 minutes of MVPA (Fig 2B). During the time spent in the MVPA intensity range the mean ± SD heart rate was 145 ± 10 b/min for the Standard group and 146 ± 12 b/min for the Incentive group (p = 0.764 for between group comparison). As expected, the total payment received for exercise sessions in Phase 1 was higher for the Incentive group than for the Standard group (Table 1).

At the end of Phase 1, both payment groups had higher absolute BMI compared to baseline, but BMI percentile and body composition were not significantly altered (Table 1). VO_2_peak increased 10% in both groups but daily steps recorded on the pedometer was not significantly changed. The improvement in aerobic fitness during Phase 1 was positively correlated with the number exercise sessions completed (r = 0.368, p < 0.01) but was not significantly correlated with session duration (r = -0.207, p = 0.163). Fasting glucose, insulin, and insulin sensitivity were unchanged. Mean HbA1c increased slightly but significantly within the Standard group.

### Phase 2

At the end of Phase 1, five girls and two boys were involuntarily withdrawn from the study because they did not complete at least 16 exercise sessions (Fig 1). Their mean age (14.9 ± 1.1 y) and BMI (37.9 ± 7.0 kg/m^2^, 99 ± 1 percentile) were not different from the participants that completed 16 or more sessions in Phase 1. Thus, at the start of Phase 2, there were 51 remaining participants who were randomized, five of whom chose to withdraw before starting to exercise. Twenty-three girls and 23 boys completed at least one exercise session in Phase 2 (range 1–61 sessions). Twelve girls and 14 boys withdrew voluntarily before the end of Phase 2, but their exercise behavior was available for analysis. The characteristics of the participants who voluntarily withdrew during Phase 2 (Age: 14.0 ± 2.5 y; BMI: 36.3 ± 6.3 kg/m^2^, 98 ± 3 percentile; exercise sessions completed in Phase 1: 35 ± 11) were not different from those of participants who completed Phase 2 (Age: 14.8 ± 2.4y; BMI: 32.9 ± 7.3 kg/m^2^, 97 ± 5 percentile; exercise sessions completed in Phase 1: 37 ± 13). As expected, though, the participants who withdrew during Phase 2 completed fewer exercise sessions (10 ± 7, range 1 to 27 sessions) than participants who finished Phase 2 (27 ± 16, range 3 to 61 sessions, p < 0.001 for between group comparison).

Consistent with the hypothesized outcome for Phase 2, the cumulative MVPA time for Phase 2 was 55% greater in the Incentive versus the Standard group (Table 2). Although the between group comparison for total MVPA time did not reach statistical significance, the confidence interval contains a lower bound close to zero and an upper bound that suggests a markedly better exercise behavior in the Incentive group (95% CI = -0.6 to 9.5 hours; p = 0.087). Moreover, the higher financial incentive resulted in longer exercise sessions (Table 2). The Incentive group had 8.3 minutes more MVPA per session than the Standard group (95% CI: 3.3 to 13.2; p = 0.002). This difference persisted even if dropouts or participants who averaged less than 1 session per week were removed from the analysis. Participants in the Standard group in Phase 2 completed 14% less MVPA time per session than they did during Phase 1 (-5 ± 7 minutes per session, p = 0.006 for comparison with Phase 1 time per session). In contrast, those in the Incentive group increased their mean MVPA time per session by 14% (5 ± 11 minutes per session, p = 0.035 for comparison with Phase 1 time per session). The portion of MVPA time spent in the moderate intensity range did not differ between groups, or from Phase 1. The average HR during MVPA time was not different between groups (Standard: 143 ± 10 b/min, Incentive: 146 ± 12 b/min, p = 0.872 for comparison between groups). The number of qualifying sessions with at least 20 minutes of MVPA in Phase 2 did not differ between groups (Table 2) and sessions with less than 20 minutes of MVPA comprised 4.8% of all sessions recorded (also not different between groups). Compared to Phase 1, participants in Phase 2 collectively completed 50% fewer exercise sessions and 47% less cumulative MVPA time (both p < 0.01). There were no differences between groups in the number of weeks with zero to four-or-more exercise sessions (Fig 2C). However, the Incentive group had higher percentages of sessions with at least 40 minutes, and 60 minutes of MVPA, respectively (Fig 2D). Accordingly, the total payment received for exercise sessions in Phase 2 (Table 2) was higher for the Incentive group than the Standard group. There were no significant changes during Phase 2 in body composition, aerobic fitness, daily steps, or for fasting glucose, insulin, insulin sensitivity, or HbA1c (Table 2).

**Table 2:** Phase 2 clinical and physiological characteristics, and exercise performance.

	Standard Group		Incentive Group	
	Pre	Change at End of Phase	Pre	Change at End of Phase
BMI, kg/m^2^	33.6 ± 6.8	0.0 ± 1.4	34.6 ± 6.9	1.3 ± 4.2
BMI, percentile	97 ± 4	0 ± 2	98 ± 3	0 ± 1
Body fat, %	42.6 ± 6.3	-1.1 ± 3.9	44.0 ± 6.8	-0.8 ± 4.5
Fat-free mass, kg	49.9 ± 9.4	0.9 ± 2.7	51.6 ± 11.6	0.9 ± 2.9
Waist circumference, cm	103 ± 15	-2 ± 5	105 ± 16	2 ± 5
VO_2_peak, ml/kg FFM/min	33.9 ± 6.2	4.6 ± 8.9	39.0 ± 9.7	-0.7 ± 6.7
Steps per day	5,265 ± 2,199	-115 ± 2,641	6,042 ± 2,508	-2,078 ± 2,599
Glucose, mmol/l	5.1 ± 0.4	0.0 ± 0.3	5.2 ± 0.4	0.0 ± 0.4
Insulin, pmol/l	110.0 ± 80.3	2.8 ± 77.8	114.7 ± 67.5	24.6 ± 75.1
iHOMA2 (%S)	67.3 ± 34.8	-5.9 ± 62.2	62.2 ± 31.4	-6.2 ± 34.5
HbA1c (%)	5.3 ± 0.2	0.2 ± 0.2	5.5 ± 0.2	0.0 ± 0.2
Exercise sessions performed	—	16 ± 11	—	19 ± 17
Total MVPA time, h	—	8.0 ± 5.5	—	12.4 ± 11.1
MVPA time per exercise session, minutes	—	29 ± 6	—	38 ± 11§§
MVPA time in moderate intensity range, %	—	63 ± 18	—	62 ± 22
Payments for exercise, USD$	—	64 ± 43	—	106 ± 89§

Values shown as mean ± SD. Pre-values correspond to measurements performed at the end of Phase 1, before Phase 2 exercise commenced. BMI, body mass index; VO_2_peak, peak oxygen uptake during aerobic fitness test; FFM, fat-free mass; iHOMA2, integrated homeostatic model of assessment; HbA1c, hemoglobin A1c; MVPA, moderate-to-vigorous activity. The Standard group had 11 girls, 9 boys; mean age 14.0 ± 2.0 years at the start of Phase 2. The Incentive group had 12 girls, 14 boys; mean age 14.6 ± 2.8 years at the start of Phase 2.

§ Difference between groups, p < 0.05.

§§ Difference between groups, p < 0.01

### Phase 3

After 5 girls were involuntary withdrawn at the end of Phase 2 for completing fewer than 16 exercise sessions in Phase 2, the 15 remaining participants were randomized for Phase 3 (S1 Fig). One girl and one boy chose not to start the exercise program in Phase 3, while three boys withdrew before the end. As was the case in Phases 1 and 2, the characteristics of participants who withdrew from the study did not differ from those who completed Phase 3. We recorded exercise behavior, but due to the unexpectedly low sample size in Phase 3, we did not conduct statistical comparisons between the groups or analyze the effects of exercise on secondary outcomes. The Ramp-down group completed 15 ± 2 exercise sessions (range 13–18) with 40 ± 10 minutes of MVPA per session. The Raffle group completed 14 ± 7 exercise sessions (range 2–24) with 39 ± 12 minutes of MVPA per session. Collectively, the participants in Phase 3 completed 20 fewer exercise sessions, on average, than they did in Phase 2 (35 ± 14 sessions in Phase 2, p < 0.01) but their MVPA time per session was unchanged (37 ± 11 minutes in Phase 2, p = 0.230). The cumulative MVPA for Phase 3 (554 ± 259 minutes per participant) and payment for exercise sessions ($53 ± 34) were less than in Phase 2 (1,276 ± 582 minutes, p = 0.001; $172 ± 79, p = 0.001), but the distribution of MVPA time spent in the moderate intensity range (60 ± 23%) remained unchanged from Phase 2.

### Predictors of exercise behavior

We used questionnaires and telephone interviews to identify perceived barriers and other reasons that participants had low compliance or withdrew from the study before finishing Phase 3. Many of the participants who withdrew were either lost to follow-up or did not cite specific reasons for their discontinuation. Among those who responded to questionnaire items, time demands from work, school, or other obligations, lack of transportation, illness, and relocation were the most common reasons cited for withdrawal from the study, while the provision of money and interest in improving physical condition were frequently cited reasons for initial interest. None of the participants stated that payments were too low to be valued.

Bivariable and multivariable regression analyses were performed to determine if the participants' descriptive and physiological characteristics were associated with their exercise behavior (number and duration of exercise sessions, total MVPA time). In Phase 1, the pool of predictor variables explored included sex, and baseline age, Tanner stage, body mass, BMI, body fat and fat-free mass, aerobic fitness, and physical activity (steps per day). In bivariable analyses, the number of exercise sessions completed in Phase 1 was inversely correlated separately with age (Spearman's r_s_ = -0.305, p = 0.007) and Tanner stage (r_s_ = -0.394, p < 0.001). In a stepwise multivariable model, only Tanner stage remained as a significant predictor of the number of exercise sessions completed, indicating that as maturation increased, frequency of exercise decreased. No combinations of two or more variables more fully explained the variance in the number of completed exercise sessions. In Phase 1 the girls were older (15.1 ± 0.4 y) than the boys (13.4 ± 0.3 y, p < 0.001) and had more advanced Tanner stage (3.7 ± 0.1 versus 2.6 ± 0.2, p < 0.001). The number of completed exercise sessions was higher in boys (31 ± 3 sessions) than girls (23 ± 2, p = 0.029) but the time per exercise session did not differ (boys = 33 ± 7 minutes/session, girls = 34 ± 8 minutes/session, p = 0.533). A comparison of less mature (Tanner stages 2 and 3; n = 42) and more mature participants (Tanner stages 4 and 5; n = 35) revealed that those who were less mature completed more exercise sessions in Phase 1 (32 ± 14) than the higher Tanner stage group (21 ± 14, p < 0.001) but the time per exercise session was not different (32 ± 7 versus 34 ± 8 minutes/session, respectively, p = 0.349). Since sex, age, and Tanner stage were highly correlated with one another it is unsurprising that we found no useful multivariable models to explain the variation in exercise frequency.

A similar statistical approach was used for Phase 2 to assess predictors of exercise behavior. Neither the number of completed exercise sessions, nor the duration of exercise sessions, were significantly correlated with age, body mass, BMI, body fat and fat-free mass, aerobic fitness, or physical activity measured at the start of Phase 2. As in Phase 1, the girls in Phase 2 were older than the boys (15.4 ± 2.6 versus 13.3 ± 1.8 y, respectively, p = 0.004). Girls also had a tendency to complete fewer exercise sessions than boys (14 ± 10 versus 21 ± 17, p = 0.090), but exercised longer per session (37 ± 10 versus 31 ± 9 minutes, p = 0.033). The only variable that correlated significantly with the number of exercise sessions in Phase 2 was the number of exercise sessions completed in Phase 1 (r = 0.624, p < 0.001).

Next, we compared the descriptive characteristics and exercise behavior of the 13 participants in Phase 3 (completers) to the other 64 participants who withdrew in Phases 1 and 2 (non-completers). Completers and non-completers did not differ in baseline age, body composition, or aerobic fitness. Baseline physical activity among completers was 8,886 ± 4,231 steps per day, and among non-completers was 5,941 ± 2,417 steps per day (95% CI = 207 to 5,683 steps/d; p = 0.058). This difference in physical activity persisted at the end of Phase 1 (completers: 7,526 ± 2,404 steps per day, non-completers: 5,655 ± 2,910; 95% CI = -74 to 3,617 steps/d; p = 0.078), and Phase 2 (completers: 6,680 ± 3,738 steps per day, non-completers: 3,934 ± 998; 95% CI = -148 to 5,640 steps/d; p = 0.061). In Phase 1 the completers recorded more exercise sessions (43 ± 11 versus 24 ± 14 sessions; 95% CI = 12 to 26 sessions; p <0.001), and spent more of their time in the vigorous intensity range (48 ± 20% versus 34 ± 17% of MVPA duration was vigorous intensity; 95% CI = 2 to 25%; p = 0.035), though their duration per session did not differ from the non-completers (35 ± 7 versus 33 ± 8 minutes, p = 0.22).

### Impact of protocol modifications and adverse events

As noted, the inclusion criteria for the study were changed about halfway through the recruitment period, with the goal of increasing enrollment by increasing the ranges for age and BMI percentile. However, only nine additional participants, all girls, were enrolled because they met the broader inclusion criteria for BMI in the overweight range (n = 4), age 18.0 to 20.9 y (n = 3), or both lower BMI and higher age (n = 2). Four of those nine participants withdrew during Phase 1, and the other five withdrew during, or at the end of Phase 2. Removing the results of those nine participants did not significantly alter the main outcomes of the study. Since we found that the number of exercise sessions completed in Phase 1 was negatively correlated with age and Tanner stage, we repeated that analysis after removing the five girls who were older than 18 y to determine if they disproportionately influenced that result. However, removing their results had no appreciable impact, as the number of exercise sessions in Phase 1 remained negatively correlated with Tanner stage (r_s_ = -0.405, p < 0.001) and age (r_s_ = -0.333, p = 0.004).

There were no adverse events attributable to participation in the exercise program. Three participants were diagnosed with T2D at the end of Phase 1. Two of those participants were ineligible to continue to Phase 2 because they completed only 7 or 8 exercise sessions, respectively. The third participant diagnosed with T2D voluntarily withdrew after completing 4 exercise sessions in Phase 2 and was not available for follow-up.

## Discussion

In this study, we explored whether carefully designed financial incentives could increase physical activity in previously sedentary overweight/obese, American Indian adolescents. This strategy was developed so that the targeted population would improve their health status and lower their future risk for diabetes and other cardiometabolic diseases. The program was partly successful. Most participants were initially enthusiastic about engaging in exercise and receiving payments for their efforts. Study participants collectively completed 3,229 exercise sessions and improved their aerobic fitness in Phase 1. In Phase 2, the enhanced incentive to increase the duration of exercise sessions appeared to work as hypothesized. However, the higher incentive in Phase 1 did not promote an increase in exercise frequency. Additionally, the payments did not provide a reward strong enough to sustain the exercise behavior, as demonstrated by the high rates of withdrawal from the study after the first 16 weeks. Throughout the study’s three phases, the average duration of MVPA time was ~34 minutes per exercise session, but the participants completed, on average, only half of the targeted number of three sessions per week. Thus, the participants were challenged to consistently attend the wellness center. However, once at the center, they had a high likelihood of completing a meaningful volume of exercise.

The rate of adherence with the exercise program goal in the current study was lower, and the rate of participant withdrawal from the study was higher, than in some comparable exercise interventions performed with overweight/obese youth [11, 12, 30]. The reason for higher adherence and retention in those studies might be because they featured a more structured, supervised exercise program for only 12–13 weeks, and in at least one case [12], transportation was provided to the community center where group activity classes were performed. Other groups have demonstrated the challenge of keeping young people with low physical activity engaged in longer exercise trials. In the HEALTHY study [13], a 22-week, supervised exercise intervention with 304 obese adolescents, participants completed ~60% of the prescribed exercise sessions, and 24% of participants withdrew before completion, numbers that are similar to Phase 1 of the current investigation. Furthermore, the HEALTHY study used a preliminary four-week run-in period to exclude participants with low initial adherence. That step eliminated 54 people (15% of the enrolled cohort), so adherence rates in the main intervention phase could have been even lower had those participants been allowed to continue. Interestingly, the authors of the HEALTHY study speculated that exercise adherence could have been higher had they used financial incentives [13].

Although studies in adults support the potential value of financial incentives to reinforce exercise behavior [17–22], to our knowledge, only one prior investigation reported the use of financial incentives to promote specific health behaviors in children or adolescents [23]. In each of those prior studies, the targeted behavior was either self-directed walking or exercise at a nearby fitness center, and the participants had a wide range of baseline health status and physical activity levels. The novel aspect of the current project was the attempt to elicit and reinforce exercise behavior in AI youth who live in a predominantly rural area and with high diabetes risk, due to their overweight/obesity and low habitual physical activity.

We used financial incentives because studies with adults suggested that better exercise adherence might be achieved with monetary versus other types of incentives [19, 21, 31–34]. Incentives such as specific gifts are likely to have variable appeal and reinforcement value among participants. Some behavior modification studies with obese children restricted television time at home with special video units controlled by parents and investigators, and then used increased viewing time a reward for physical activity [35–37]. That strategy may be too expensive and difficult to implement on a large scale. In comparison, financial reinforcement of behavior is simple to implement and broadly applicable. We used a reloadable debit card for each participant, which allowed the frequent distribution of variable amounts of money based on exercise behavior.

We did not include unpaid participant groups since most research participants, in our experience, expect compensation for time and travel. Thus, we cannot determine the effect of financial incentives *per se* on exercise behavior. However, since none of the participants were previously using the wellness centers before entering the study, the incentives appear to have promoted at least a transient increase in exercise behavior in the majority of participants. We were sensitive to the need to balance the use of money to elicit high adherence rates with the ethical concern that overly large payments could result in undue influence. Thus, the payments were selected to be commensurate with amounts used in our recent and ongoing studies with children, which use similar procedures and require similar time commitments as in the current investigation. The total time for testing and exercise sessions per participant (assuming 100% compliance) was up to ~164 hours over ~50 weeks, not including transportation time between the participant’s home or school and the wellness centers and testing sites. Depending on group assignment, the range of total payments available to participants who completed all assigned study visits was approximately $716 to 1,292, or about $4.40 to 7.90 per hour of involvement. This is similar to the hourly wage an adolescent living in this region could earn at a part-time job, and similar to, or greater than incentives for exercise in prior studies with adults or children [19–23]. In is unclear whether higher amounts of money would elicit more frequent exercise or longer retention in the exercise program, but larger incentives could be difficult to support within the budgets of wellness organizations and could limit generalizability.

A potential concern when using financial incentives is that they may undermine the targeted behavior, especially once the incentives are removed. This effect has been demonstrated in adults performing simple tasks [38] or short-term diet and physical activity interventions [39, 40]. The authors of those studies suggested that participants may perceive payments for a specific behavior to be controlling, and the elimination of payments might decrease motivation to maintain the behavior. In contrast, a recent study conducted with college students demonstrated that payments for exercise did not result in a reduction in either intrinsic or extrinsic motivation to perform physical activity over nine months [22]. Likewise, two literature reviews concluded that, on balance, there was a lack of evidence that the provision of financial incentives consistently reduce intrinsic motivation of people to perform healthy lifestyle behaviors, especially for people like those in the current investigation with low baseline levels of the targeted behavior [41, 42].

The financial incentives in the current study were based on task-completion (payment per exercise session) rather than achieving a specific performance outcome (e.g., improving aerobic fitness or insulin sensitivity), but we did not measure the impact of the payments on motivation. Phase 3 was designed to determine whether the exercise behavior would persist when the payments were withdrawn or made infrequent. The majority of participants withdrew before Phase 3 so we cannot determine whether decreasing the financial incentives undermined exercise performance or participation. However, since the exercise payments were still available when participants withdrew, it appears likely that other factors (e.g., competing time demands from school, work or friends, lack of transportation, desire for additional social support at the fitness center), were responsible for the decline in participation over time. Thus, additional strategies are needed to keep adolescents engaged in an exercise program once they begin.

Although our study was not designed to test for differences in exercise adherence between boys and girls, exploratory analyses suggested that they might differ. In Phases 1 and 2 boys completed more exercise sessions than girls, though the boys were also younger and had a lower Tanner stage at baseline. The finding that boys were more active is consistent with many studies of objectively measured free-living physical activity in adolescent boys and girls [2, 3]. Likewise, the finding in Phase 1 that exercise frequency was lower among older participants and those in Tanner stages 4 and 5 is consistent with the finding that physical activity tends to decline during adolescence [2, 3]. This makes creating exercise programs for this age group that are attractive and sustainable important, but difficult. A key finding was that exercise duration per session in Phase 1 did not differ with sex, age, or maturation stage. This supports the general finding that specific behavioral strategies are needed to help this population of adolescents to initiate physical activity sessions; once adolescents reached a wellness center, they demonstrated a willingness to perform a meaningful duration of activity regardless of their age or sex. There were no clearly defining characteristics of the small cohort of participants who completed the entire protocol, but they appeared to have higher free-living physical activity at baseline and throughout the study, and they spent more of their exercise time in the vigorous intensity range. This subset of "completers" may have therefore been more prepared for the intervention. Future studies will need to address the barriers that prevented more participants from reaching the exercise targets.

A secondary goal of the study was to measure the impact of the exercise program on aerobic fitness, body composition, and insulin sensitivity. Aerobic fitness was improved 10% after the first 16 weeks of exercise, but did not significantly increase thereafter, and insulin sensitivity and body composition were unchanged throughout the study. Thus, the exercise had a modest effect on improvement of metabolic health and decreasing the risk for developing diabetes. The importance of mitigating metabolic disease risk in this population was underscored by the fact that three participants were diagnosed with T2D at the end of Phase 1. Several previous studies demonstrated that 10–22 weeks of supervised exercise on 3–5 days per week can result in increased aerobic fitness and decreased insulin resistance and abdominal fat in overweight/obese children and adolescents [12, 13, 43, 44]. Davis et al., [12] reported several health and fitness benefits that occurred in response to only 20 minutes per day of aerobic activity, although a key to that finding may be that the exercise was conducted on five days per week (i.e., 100 minutes per week) for 13 weeks, and protocol adherence was 85%. Compared to those prior studies [12, 13, 43, 44], the participants in the current investigation exercised less frequently (~1.7 sessions per week in Phase 1) and accrued a lower total volume of MVPA (~56 minutes per week in Phase 1). This relatively low level of physical activity likely explains the lack of significant change in clinical outcomes other than aerobic fitness. Since the incentives in the current study were successful at increasing exercise duration, our future goal is to target ways to increase exercise frequency, to increase the likelihood of increasing insulin sensitivity and to reduce the risk for diabetes in this population.

## Conclusion

Financial incentives were modestly successful in promoting exercise behavior in previously sedentary AI adolescents at risk for diabetes. The incentives appeared to help promote the initial exercise behavior and in the study's second phase participants responded to enhanced incentives by increasing the duration of their exercise sessions. The exercise program also resulted in an increase in aerobic fitness. However, the incentives did not promote higher exercise frequency as expected, and retention in the program was low. It remains unclear whether greater financial incentives would have resulted in even greater exercise intensity or increased frequency, or whether incentive-induced exercise behavior is sustained or reduced after such incentives are withdrawn. It is clear, however, that resistance to lifestyle change in obese, sedentary adolescents is substantial [45] and that novel strategies to influence exercise behavior in this group of children likely will be required.

## Supporting information

S1 CONSORT Checklist CONSORT 2010 checklist of information to include when reporting a randomized trial. (PDF)

S1 Protocol Incentivizing behavior: Promoting more physical activity in American Indian youth. Research protocol for the study.
(PDF)

S1 Table Payments for Phase 3. Participants in Phase 3 (Weeks 33–48) were randomly assigned to either the Ramp-down or Raffle group. The Ramp-down group earned progressively less money each week. The bonus was provided to participants who completed three sessions in the corresponding week. For the Raffle group, the number of chances increased with both frequency and duration of exercise. Exercise sessions with less than 20 minutes of moderate-to-vigorous physical activity did not earn a payment or raffle chance, respectively.
(PDF)

S1 Fig Flow chart showing the number of participants in Phase 3. The Ramp-down group received diminishing payments while the Raffle group received discontinuous payments, as described in the text.
(PDF)
