# Correction: Using financial incentives to promote physical activity in American Indian adolescents: A randomized controlled trial

**DOI:** 10.1371/journal.pone.0231075

**Published:** 2020-03-25

**Authors:** Kevin R. Short, Jennifer Q. Chadwick, Tamela K. Cannady, Dannielle E. Branam, David F. Wharton, Mary A. Tullier, David M. Thompson, Kenneth C. Copeland

There is an error in the footnote of Table 1. “Increase from baseline to end of Phase 1 within group, p < 0.03” should be corrected to “Increase from baseline to end of Phase 1 within group, p<0.05”. Please see the correct Table 1 here.

**Table 1:** Clinical and physiological characteristics at baseline, changes at the end of Phase 1, and exercise performance in Phase 1.

	Standard Group		Incentive Group	
	Baseline	Change at End of Phase	Baseline	Change at End of Phase
BMI, kg/m^2^*	34.9 ± 8.5	0.7 ± 1.1†	34.7 ± 6.2	0.7 ± 1.8†
BMI, percentile	98 ± 3	0 ± 1	98 ± 3	0 ± 1
Body fat, %	43.6 ± 8.1	0.9 ± 2.7	43.1 ± 6.9	0.1 ± 4.2
Fat-free mass, kg	48.6 ± 10.2	0.6 ± 2.9	52.2 ± 10.2	0.9 ± 4.3
Waist circumference, cm	104 ± 16	-1 ± 5	108 ± 12	0 ± 8
VO_2_peak, ml/kg FFM/min*	34.2 ± 7.1	3.4 ± 5.7†	35.2 ± 8.7	3.7 ± 7.5†
Steps per day	6,404 ± 3,425	-869 ± 2,318	6,218 ± 2,419	483 ± 3,401
Glucose, mmol/l	5.3 ± 0.5	0.2 ± 1.0	5.1 ± 0.4	0.4 ± 2.1
Insulin, pmol/l	164.7 ± 229.3	-2.9 ± 166.9	119.7 ± 94.3	-11.4 ± 80.3
iHOMA2 (%S)	74.9 ± 48.5	-13.8 ± 40.7	68.6 ± 44.2	-4.9 ± 26.2
HbA1c (%)	5.4 ± 0.3	0.1 ± 0.2†	5.3 ± 0.2	0.1 ± 0.6
Exercise sessions performed	—	26 ± 16	—	28 ± 15
Total MVPA time, h	—	15.2 ± 10.1	—	15.0 ± 8.1
MVPA time per exercise session, minutes	—	35 ± 7	—	32 ± 8
MVPA time in moderate intensity range, %	—	63 ± 18	—	64 ± 18
Payments for exercise, USD$	—	99 ± 58	—	245 ± 144§

Values shown as mean ± SD. BMI, body mass index; VO_**2**_peak, peak oxygen uptake during aerobic fitness test; FFM, fat-free mass; iHOMA2, integrated homeostatic model of assessment; HbA1c, hemoglobin A1c; MVPA, moderate-to-vigorous activity. The Standard group had 18 girls, 17 boys; mean age 14.2 ± 2.4 years at baseline.

The Incentive group had 25 girls, 17 boys; mean age 14.4 ± 2.3 years at baseline.

* Main effect of time for entire cohort, p < 0.01.

† Increase from baseline to end of Phase 1 within group, p < 0.05.

§ Difference between groups, p < 0.01.

## References

[1] Short KR, Chadwick JQ, Cannady TK, Branam DE, Wharton DF, Tullier MA, et al (2018) Using financial incentives to promote physical activity in American Indian adolescents: A randomized controlled trial. PLoS ONE 13(6): e0198390 10.1371/journal.pone.0198390 29856832 PMC5983431

