# Supplementary material for: Using financial incentives to promote physical activity in American Indian adolescents: A randomized controlled trial
Source: PLoS One. 2018 Jun 1;13(6):e0198390. doi: 10.1371/journal.pone.0198390 (PMC5983431; doi:10.1371/journal.pone.0198390)
Supplement: S1 Protocol — Research protocol for the study. (PDF) [file pone.0198390.s002.pdf]

Protocol Title: Incentivizing behavior: promoting more physical activity in American Indian youth

Principle Investigators: Kenneth C. Copeland, MD[1]; Kevin R. Short, PhD[1].

Co-investigators: Jennifer Chadwick, BS[1]; April M. Teague, MS[1]; David M. Thompson, PhD, PT[2]; Mary Ayn Tullier, RN[3], Charlotte Coleman[3], Lisa Wolbert[3], Tiffanie Burchfield[3]

Affiliations: [1]Section of Diabetes/Endocrinology, Department of Pediatrics, and [2]Department of Biostatistics and Epidemiology, University of Oklahoma Health Sciences Center; 3Choctaw Nation Healthcare Services Authority, Choctaw Nation of Oklahoma

Funding Source: National Institutes of Health, National Institute on Minority Health and Health Disparities
This is Research Project 2 as part of Parent Grant: 2P20 MD000528-10 (PI: Neil Henderson, PhD, College of Public Health, OUHSC)

Human Research Participant Protection
The University of Oklahoma
IRB APPROVED
AAHRPP
IRB NUMBER: 0434
IRB APPROVAL DATE: 01/04/2016

ABSTRACT

Among youth populations, American Indians have the highest prevalence of diabetes in the United States. This study will use exercise as the principal lifestyle modification approach to reduce the risk of diabetes in this population. The Choctaw Nation of Oklahoma has several excellent, but underutilized wellness facilities in their Health Services Area in rural SE Oklahoma, a low socioeconomic region. It has been established that exercise lowers diabetes risk, and many obese, insulin resistant American Indian youth who live in this region would benefit from an increase in regular exercise. The challenge is to modify behavior so that routine exercise is established and maintained. The proposed study will test whether monetary incentives can elicit greater frequency and duration of exercise in American Indian youth when transportation and access barriers are reduced. This strategy is outlined in 3 Specific Aims to determine whether:

1. **Financial incentives will increase the frequency of exercise sessions performed by American Indian youth.** In a 16-week trial overweight or obese, insulin resistant American Indian youth, 11-21 years old, will be randomly assigned to Control or Incentive groups that differ only in the amount of money they are given for completing up 3 exercise sessions per week.

2. **Financial incentives will increase the volume of exercise performed by American Indian youth.** After completion of Aim 1 participants will be re-randomized to either a Control or Incentive group for a second 16-week exercise period. Groups will differ only in the amount of money paid for the amount of time per session spent in aerobic exercise.

3. **Ongoing incentives will be required for maintenance of fitness center utilization established with financial incentives.** Participants who have progressed through Aims 1 and 2 will continue exercising for a third 16-week period. Each participant will be re-randomized to either a ramp-down incentive scheme, or a raffle group that earns prize chances based on their exercise frequency and duration, to examine methods influencing durability of exercise behavior.

As an additional goal **(Aim 4)**, we will **measure the impact of physical activity and physical fitness on circulating biomarkers of metabolic disease risk**. Normal weight American Indian youth with either low physical activity and fitness or high physical activity and fitness will be studied on a single occasion. Serum/plasma concentrations of lipids, amino acids, and related markers of inflammation, and oxidative and vascular stress will be compared among the normal weight participants and the overweight/obese youth enrolled in Aims 1-3.

This study will result in an increased understanding of how to promote exercise in youth at high risk for cardiometabolic disease. A secondary goal is to determine how diabetes risk factors vary with exercise volume and fitness. These data will be of interest to health care providers to American Indians, and to all youth, as well as to public policy makers.

Human Research Participant Protection
The University of Oklahoma
IRB APPROVED
IRB NUMBER: 0434
IRB APPROVAL DATE: 01/04/2016

SPECIFIC AIMS

Half of adolescents in the United States fail to reach recommended levels of physical activity and nearly a third are overweight, elevating cardiometabolic risk early in life and setting the stage for adult disease. American Indians have the highest prevalence of type 2 diabetes in the United States, an elevated risk that is evident even in childhood. Thus, effective interventions are needed to reduce the lifetime medical and socioeconomic burdens of diabetes and related diseases. Through our prior clinical and research partnerships with American Indians in Oklahoma, we have gained insight into the health concerns of American Indian youth and the importance of community and healthcare leaders in developing creative collaborative approaches that work towards solutions. Based on this expertise, we developed the proposed strategy to improve the health of sedentary, obese American Indian youth by providing incentives for increased physical activity.

The high-risk youth who will be enrolled in the proposed study live in a predominantly rural, low socioeconomic region of Oklahoma. They currently have access to several well-equipped fitness centers in the region, but these resources are underutilized. Although it is well-accepted that regular exercise can improve individual health and reduce the socioeconomic burden of diabetes, reducing barriers and effectively modifying behavior presents a significant challenge. Prior studies have shown that paying adults for desired behaviors and/or health outcomes can be effective, but the use of financial incentives to shape exercise behavior in adolescents has not been reported. **We hypothesize that monetary incentives will encourage obese, insulin resistant American Indian youth to establish and maintain better exercise habits, ultimately resulting in better health.** We propose to provide transportation for participants to the wellness centers and individually tailored instruction and oversight by fitness professionals so that the centers are accessible and appealing. The provision of monetary incentives is designed to reinforce the frequency and duration of exercise. Our strategy to accomplish these goals is outlined in the following Specific Aims:

Aim 1: To determine whether financial incentives will increase the **frequency** of exercise sessions performed by American Indian youth at a community fitness center. Obese, insulin resistant American Indian adolescents will be randomly assigned to one of two groups for a 16-week exercise program: Control ($4/session, up to 3 sessions/week, provided as compensation for time), or Incentive ($4, $10, and $16 for the first, second, and third session each week, respectively). All participants will be provided transportation to and from the fitness center and professional instruction and supervision for aerobic exercise training, but will differ only in the amount of money they are eligible to collect for their attendance. A secondary goal of this Aim is to assess the association between exercise frequency and changes in diabetes-related risk factors, including insulin sensitivity, body composition, and blood lipids.

Aim 2: To determine whether financial incentives will increase the **duration** of exercise performed by American Indian youth at a community fitness center. Participants who complete Aim 1 will be re-randomized to one of two groups for a second 16-week period of exercise: Control ($4/session, up to 3 sessions/week), or Incentive ($4, $7, or $10 for 20-, 40-, or 60- minute sessions, respectively, up to 3 sessions and $30/week). Transportation and professional supervision will remain the same as in Aim 1. A secondary goal is to assess the association between exercise duration and changes in diabetes-related risk factors.

Aim 3: To determine whether ongoing incentives are required for **maintenance** of fitness center utilization established with financial incentives. Participants who complete Aim 2 will enter a third 16-week exercise period with diminished monetary incentives. Each person will be re-randomized to one of two incentive structures: a) Ramp-down, with financial incentives diminishing to zero by 8 weeks, or b) Raffle, with monetary prizes awarded through a drawing in which chances of winning are dependent on frequency and duration of exercise. We hypothesize that exercise behavior will remain above zero even after financial incentives decline and that the Raffle approach will reinforce the best exercise behavior maintenance. Clinical diabetes risk outcomes will be measured as in Aims 1 and 2.

Aim 4: To measure the impact of physical activity and physical fitness on circulating biomarkers of metabolic disease risk. Normal weight American Indian youth with either low physical activity and fitness or high physical activity and fitness will be studied on a single occasion. Serum/plasma concentrations of lipids, amino acids, and related markers of inflammation, and oxidative and vascular stress will be compared among the normal weight participants and the overweight/obese youth enrolled in Aims 1-3.

This study will clarify how specific incentive structures can be used to promote exercise in a population of adolescents at exceptionally high risk for diabetes and cardiometabolic disease. The study will also provide needed insight on the volume of exercise needed to elicit changes in clinical outcomes. These results will be

Human Research Participant Protection The University of Oklahoma IRB APPROVED AAHRPP
IRB NUMBER: 0434
IRB APPROVAL DATE: 01/04/2016

used to develop better lifestyle intervention strategies for American Indian youth, and for other predominantly rural, lower socioeconomic status people faced with the distress of elevated diabetes risk. This study will provide valuable data for clinicians who treat American Indians and youth in general, as well as for public policy makers.

RESEARCH STRATEGY PART A: SIGNIFICANCE

Prevalence and socioeconomic burden of obesity and diabetes: Over the past 40 years, childhood obesity in the U.S. has increased 4-fold, and ~30% of children are now overweight or obese (BMI ≥ the 85th percentile for age and sex) (69, 70). The serious social, economic, and public health costs of childhood obesity and sedentary lifestyle are recognized as national health priorities. In 2006 the US Government Accounting Office reported that health care expenditures for obese children are ~3 times higher than average (42). In addition, obesity contributes to the growing prevalence of diabetes (~9-13% of all Americans) and pre-diabetes (16% of youth and 30% of adults) (29, 30, 61). While the prevalence of **type 2 diabetes (T2D)** is highest in older adults (29), ~200,000 youth ≤19 years of age now have T2D (35, 82, 108), and metabolic syndrome is present in 25-50% of obese adolescents (59). The effects of obesity and low physical activity during adolescence are compounded by their association with dyslipidemia and hypertension and likely persistence into adulthood (37, 67, 89). Thus, the alarming rates of childhood obesity now will only exacerbate the socioeconomic costs of diabetes and related metabolic disorders in the future. These data highlight the urgent need for novel approaches to the problem of childhood overweight and obesity and associated health complications.

Association of obesity with cardiometabolic disorders: Insulin sensitivity, a recognized marker of metabolic risk, is impaired in people who are overweight or obese and improved by exercise training. Overweight or obese children are more likely than their normal weight peers to have insulin resistance, impaired beta-cell function and elevated cardiometabolic risk factors such as dyslipidemia, and hypertension (87, 108, 110, 111). Insulin resistance is also associated with increased concentrations of circulating inflammatory markers (96, 97, 109) and lower adiponectin (20). Growing evidence links inflammation (e.g., adipocytokines) with abdominal adiposity and insulin resistance (51), and abdominal adiposity is a strong predictor of insulin resistance (14, 56, 85). Thus, we have targeted children with insulin resistance for the current study because of their elevated risk of developing diabetes, and we will use insulin sensitivity as one of the clinical outcomes for measuring the effects of exercise on health.

The problem of diabetes in American Indians and the Oklahoma partnership motivating this proposal: The prevalence of T2D has become a leading health concern for American Indian people, as prevalence of T2D (13.1%) among American Indians is higher than in any other racial/ethnic groups in the U.S. (93, 94). The disproportionate burden of T2D in American Indians was shown in the SEARCH for Diabetes study, which estimated the prevalence of diabetes in children from several racial/ethnic groups (82). In youth 10-19 years old, T2D accounted for 76.4% of diabetes cases in American Indians but only 5.8% of cases in their non-Hispanic white peers. These and other data clearly implicate genetic and/or environmental factors in the elevated T2D risk seen in American Indian youth from an early age. Therefore, effective intervention programs are urgently needed to reduce diabetes risk in this population. The relative benefits of intervention programs may be most discernible in a high-risk population.

The participation of American Indian tribes, including the Choctaw Nation of Oklahoma, in recent clinical research trials demonstrates the commitment and resolve of Oklahoma tribes to address health problems. For the last 7 years, Oklahoma has been the leading recruitment site nationally for the TODAY (**T**reatment **O**ptions for **D**iabetes in **A**dolescents and **Y**outh) study, a multi-centered trial sponsored by NIDDK that is designed to define optimal treatment of T2D in youth (28, 98). The Choctaw Nation has been a major and effective partner in these efforts. Nevertheless, the obesity and diabetes epidemics continue in Oklahoma and nationwide. Our recent and ongoing experience in Oklahoma in the TODAY trial taught us that many barriers to successful intervention must be eliminated to reduce the diabetes epidemic (28, 107), particularly for youth living in rural parts of Oklahoma. These barriers include high rates of poverty, low rates of any family member obtaining a high school education, and high consumption of high-calorie, nutrient-poor foods. Due to lack of transportation and other social complexities, families living in rural areas of the state often fail to attend their scheduled medical clinic visits. <u>However, we have experienced superior adherence and attendance for study</u>

Human Research Participant Protection
The University of Oklahoma
IRB APPROVED
AAHRPP Association for the Accreditation of Human Research Protection Programs, Inc.
Full AAHRPP Accreditation
IRB NUMBER: 0434
IRB APPROVAL DATE: 01/04/2016

visits in the TODAY trial compared to prior research studies, because guaranteed, reliable transportation and incentives for participation were provided (88).

The recent experiences of the TODAY trial have shown that preventing T2D in American Indian youth requires novel approaches that overcome social barriers. This may require major changes in public health policy that extend beyond making exercise facilities and healthier foods more readily available. The success of the TODAY trial in Oklahoma demonstrates the strong commitment that American Indian leaders are making in the effort to understand and eliminate the health disparities facing American Indian youth. Since the start of the TODAY trial in 2003, Greg Pyle, Principal Chief of the Choctaw Nation, has publicly championed efforts to reduce obesity among American Indian youth and consistently supported improved health policies and preventative programs. This vested involvement of community leaders like Chief Pyle and the clinical and wellness center staffs will contribute to the successful completion of the current proposal. A recent review of lifestyle intervention programs to prevent obesity and diabetes in American Indian communities highlighted the need for such commitments from community leaders (43). The TODAY trial indicates that the increasing rates of obesity and T2D in American Indian youth can be reversed if barriers to participation are overcome. Therefore, although we will obtain valuable information regarding the effects of exercise on metabolic outcomes, the primary focus of this proposal is to define effective incentives for increasing participation of at-risk youth in an exercise program, after barriers to access have been reduced. The knowledge gained through this study will be used to improve diabetes prevention programs and to shape public health policies.

The Choctaw Nation of Oklahoma's experience of health distress: The Choctaw Nation of Oklahoma has implemented preventative programs to reduce health disparities. However, current health statistics demonstrate that further improvements are needed to reach the benchmarks for success. In 2010 Choctaw Nation Health Service published a report on the health status of the people in their service area of SE Oklahoma (24) using data compiled from their Resource and Patient Management System, the Oklahoma State Department of Health, and the U.S. Census Bureau. Several key data from this report highlight the disproportionate health and socioeconomic concerns of our target population in this study:

- The prevalence of morbid obesity (body mass index ≥ 40 kg/m^2) in the patient population of the Choctaw Nation Health Care System is 10%, which is twice the Oklahoma state average of 4.8%.
- More than 25% of girls and 30% of boys ages 12-19 years old are obese, remarkably higher than the ~18% obesity rate among all U.S. children.
- T2D prevalence has risen to 12.5% in the Choctaw Nation Health Services patient population, and diabetes is the third-leading cause of death, exceeded only by heart disease and cancer.
- Poverty rates are 17-26% in all 11 counties of the Choctaw Nation Services Area, which exceeds state (15.8%) and U.S. (13%) average rates. More than 25% of children ≤18 years old live in poverty.
- The median annual household income for this population is ~$31,000, substantially lower than the averages for Oklahoma (~$43,000) and the U.S. (~$52,000). In 9 of the 11 counties in the Choctaw Nation Services Area, unemployment rates since 2009 have exceeded the statewide average of 6.5%.

In addition to poverty, Choctaw Nation Health Service Area patients are likely to face barriers such as lack of transportation, food deserts (inconsistent food supply), and lack of safe, assessable outlets for physical activity. Several unique and potentially useful support programs are offered to help address those barriers, although there is insufficient research about community awareness, specific needs, and ultimate effectiveness of current services in reducing distress and health disparities. To improve the alignment between community needs and available programs, a structured assessment of current and future needs was conducted beginning in June 2011 by the Choctaw Nation in partnership with another academic research center, and may be available once the current project begins. Even though the focus of the current study will be on youth from the Choctaw Nation of Oklahoma, our goal is to apply the information gained and strategies developed through this proposal to other communities with similar barriers so health disparities can be adequately reduced.

Summary: Increased diabetes and cardiometabolic disease risks are looming for an entire generation of obese and largely sedentary children and adolescents in the United States. This problem is particularly acute in American Indians, many of whom face significant socioeconomic challenges. While increasing exercise participation will not address the entirety of social and clinical problems facing obese, insulin resistant youth the proposed program can establish critical progress toward improved physical and mental wellness.

Human Research Participant Protection The University of Oklahoma IRB APPROVED AAHRPP
IRB NUMBER: 0434
IRB APPROVAL DATE: 01/04/2016

RESEARCH STRATEGY PART B: INNOVATION

The use of incentives for exercise behavior: Ensuring adherence is a major challenge of behavioral intervention programs. Attempts to promote weight loss, maintenance of an exercise routine, or smoking cessation often fail because the benefits are not sufficiently obvious to the person affected (i.e., people may not fully appreciate how improving their fitness or reducing adiposity could improve their quality and length of life, particularly children). In addition, the effects of health-promoting behaviors are not immediately rewarded, and short-term temptations to deviate away from target behaviors may over-ride long-term goals (5). A financial incentive can help overcome these problems because the provision of money provides a tangible, nearly immediate reinforcement of target behavior and can offset the temptation of short-term choices that are contrary to long-term benefits. Lottery systems of monetary awards can be both successful and cost-effective because people tend to overestimate the probability of unlikely but desirable events, like winning the lottery, and therefore act to increase opportunities (54, 76). Lottery incentives have been used to promote weight loss, the completion of health questionnaires, and attendance at sports training sessions for adults (34, 44, 80, 103) and will therefore be used as a strategy to promote exercise maintenance during Aim 3 of this proposal.

Monetary incentives have been used with adults in weight loss programs, to reduce smoking and substance abuse, and to increase preventive health behaviors such as vaccinations and disease risk screening (6, 18, 41, 53, 81, 92, 95, 103). Fewer studies, however, have used monetary incentives to promote exercise behavior, and most of these were implemented in workplace settings (41, 95). Examples of studies outside of the workplace in which people were paid to exercise include an 18-month weight loss program for obese adults that showed that financial incentives and/or personal trainers resulted in significantly better exercise session attendance compared to assignment to group exercise classes (52). Another study showed that older people who could earn up to $25 per week for walking time completed more exercise than a control group that was paid a fixed amount (4.1 vs. 2.3 hours/week) (40). Finally, college students who were paid $100 to visit the campus gym 8 times in one month successfully met that goal and maintained a significantly higher frequency of gym attendance without incentives for the next 2-4 months compared to the pre-incentive period, a change that did not occur in either an unpaid control group or a group paid to visit the gym only once (23).

Although studies in adults support the potential value of financial incentives to reinforce exercise behavior, to our knowledge there are no reports on the use of financial incentives to promote specific health behaviors in children or adolescents. Therefore, eliciting and reinforcing exercise behavior in youth with high diabetes risk and living in a predominantly rural area is a highly novel aspect of the current proposal. We chose to use monetary incentives because data, albeit from studies in adults, support that better exercise adherence is achieved with financial versus other types of incentives (41, 95). Alternate incentives instead of money, such as games, sports gear, clothes or restaurant certificates, are likely to have variable appeal and reinforcement value among participants. Some behavior modification studies with obese children used restricted time for watching TV as a reward for physical activity, using special video units controlled by parents and investigators in the homes of participants (46, 47, 78). However, this strategy is expensive and difficult to implement on a large scale. In comparison, monetary reinforcement of behavior is simple to implement and broadly applicable.

The provision of monetary incentives is meant to promote and reinforce healthy behavior but for studies with youth, we appreciate the need to find an appropriate balance between using money to elicit high adherence rates and the ethical concern that undue influence could result from payments that are too large. Thus, the payments in the current investigation will be commensurate with amounts used in our recent and ongoing IRB-approved studies with children, which use similar procedures and require similar time commitments as in the current proposal. Most research studies that enroll community volunteers provide remuneration for time and travel, and many participants now expect to be paid for their efforts. By carefully structuring the relative size and schedule of payments in the current study, we seek to test whether a modest amount of money can modify healthy behaviors and, in turn, improve markers of disease risk.

Lifestyle interventions and the role of exercise: It is clear that lifestyle programs combining nutritional, behavioral and physical activity components can prevent the onset of diabetes in adults (e.g., Diabetes Prevention Program and others) (33, 38, 50, 58, 99) and improve insulin resistance and related risk factors in obese youth (e.g., Yale Bright Bodies Program and others) (9-11, 79, 84). Notably, the Diabetes Prevention Program showed that an intensive lifestyle program has a lasting impact on fasting glucose, development of diabetes and medication use for at least 7 years after the end of structured intervention (32). Lifestyle modification programs for obese children could have similar lasting benefits on healthcare costs and quality of

IRB APPROVED
IRB NUMBER: 0434
IRB APPROVAL DATE: 01/04/2016

life. However, dietary programs targeting weight loss require intensive management for success, and even then weight regain is likely (32). While family-based interventions have shown some promise for obese children, they are difficult to implement and are costly (36, 49). In addition, since many of these programs are multi-factorial, it can be challenging to parse out the relative contributions of exercise, weight-loss, or other factors to the health benefits observed (79, 84, 100), and to determine the most cost-effective ways to utilize limited financial resources.

The proposed study design will confront these practical issues and determine the effects of exercise on diabetes risk, without a targeted weight loss component or specific nutritional intervention. Recognizing the important contributions of dietary behavior to diabetes and cardiovascular risk, all participants will receive medical nutrition therapy consultations as part of their standard clinical care. After careful consideration, we chose a unique study design that does not include a dietary or weight loss intervention, in order to assess the direct effects of exercise. We also sought to develop a program that may be more cost-effective than intensive weight loss programs by incentivizing a healthy behavior (exercise) rather than a specific outcome that is difficult to achieve (weight loss). From our prior experience we are confident that we can achieve compliance with the proposed exercise program and that beneficial changes in clinical outcomes will emerge even if there is no change in body mass.

Even in the absence of weight loss, several benefits accrue with regular exercise (27). For example, increasing cardiorespiratory fitness reduces the risk of metabolic syndrome and cardiovascular disease independent of adiposity (26, 45, 60). We and others have shown that insulin resistance and abdominal adiposity can improve in adults in response to exercise training even with little or no change in body mass (17, 60, 85). Likewise, insulin sensitivity was improved in obese youth following 8-12 weeks of moderate intensity aerobic exercise despite no change in body mass (15, 68, 102). Thus, while reduction in body mass is an important goal for obese children, lifestyle interventions that increase physical activity confer significant health benefits independent of weight loss. In the current study we will measure body composition (including total and regional adiposity), insulin sensitivity, and related clinical outcomes at 16-week intervals as participants progress through the 48-week exercise training program. This will provide a unique opportunity to assess the time course of adaptations and the relative importance of exercise versus exercise plus body weight and/or body fat reduction (if and when those changes occur) on several measures of diabetes risk in youth.

Examining the effects of varied exercise volume: Current recommendations call for at least 30-60 minutes of moderate-to-vigorous physical activity (MVPA) on ≥5 days per week to maintain or improve cardiometabolic risk, fitness, and body mass in adults, and 60 minutes per day in children (7, 72, 90). Although experts acknowledge the potential dose-response benefit of greater exercise volume achieved through different combinations of exercise duration and/or intensity (7, 86), no specific guidelines are yet available to delineate the optimal quantity and type of exercise for targeting specific health goals. The recommendations for physical activity volume largely draw on epidemiological data, as few prospective intervention studies have addressed the value of different combinations of exercise duration and/or intensity (25), and no prospective studies have examined how variation in exercise volume affects insulin sensitivity in children or adolescents. The need for further data to support exercise recommendations was cited by the 2004 NIH Obesity Research Task Force (19). Although it is not the primary goal of the study, the current proposal will provide a unique opportunity to test how individual selection of exercise volume impacts several measures of cardiometabolic risk since there is expected to be a variable exercise participation rate due to the combination of personal motivation and the effect of financial incentives. Further, the study design will allow us to measure the effects of exercise frequency (Aim 1), exercise duration (Aim 2), and overall exercise volume (Aims 1-3) on clinical outcomes such as insulin sensitivity, body composition and blood lipids. These results will allow us to address the existing gaps in knowledge and to design additional interventions that further clarify this important issue.

Summary: The most novel aspects of this proposal are the examination of how financial incentives can be used to motivate exercise participation and adherence in youth and how variation of exercise volume differentially affects clinical outcomes. Existing lifestyle interventions designed to reduce obesity and improve fitness are extremely costly to our healthcare system. The proposed cost-effective approach could have high impact on public health policy if found to be effective.

IRB APPROVED
IRB NUMBER: 0434
IRB APPROVAL DATE: 01/04/2016

RESEARCH STRATEGY PART C: APPROACH

Design: Specific Aims 1-3 will follow a single prospective, randomized trial design, with 105 participants enrolled and 80-90 entering the exercise program (**Fig 2**). The individual Aims are distinguished by sequential study phases and varying incentive structures (**Fig 3 and 4**). Aim 4 is a cross-sectional comparison between overweight/obese participants entering the main trial and two groups of normal weight youth (up to 40 participants per group) with either low physical activity and fitness or high physical activity and fitness (up to 40 participants per group).

Participants: Male and female American Indian youth 11-21 years old will be recruited from the Choctaw Health Service Area of SE Oklahoma. For the primary exercise incentives study, the eligibility criteria include BMI ≥85th percentile for age- and sex-specific norms based on growth charts from the Centers for Disease Control, maturation level ≥Tanner Stage 2 for breasts (girls) or genitalia (boys), family history of T2D, and recent history of low physical activity. Low physical activity, defined as attaining <30 minutes of structured moderate-to-vigorous intensity exercise on ≤ 3 days/week over the preceding 3 months, will be confirmed through questionnaires and objective monitoring as described below. For both normal weight comparison groups in Aim 4, eligibility will include BMI greater than 20th and less than 85th percentile, maturation level ≥Tanner Stage 2 for breasts (girls) or genitalia (boys), and recent history of either a) low physical activity (same as the overweight/obese group) or, b) high physical activity (>30 minutes of structured moderate-to-vigorous intensity exercise on >3 days/week over the preceding 3 months. T2D family history will be recorded but will not be an inclusion criterion for the normal weight groups. Fitness level and physical activity will be determined through testing, described below. Additional enrollment and retention eligibility criteria are: lack of diabetes or other potentially confounding metabolic disorders, able to safely exercise, willingness to complete the testing and participation schedule, and not on medications known to impact the stated outcomes. A medical history and physical exam will be performed to assure suitability for the study during the initial screening. Inclusion and exclusion details are provided under Human Subjects.

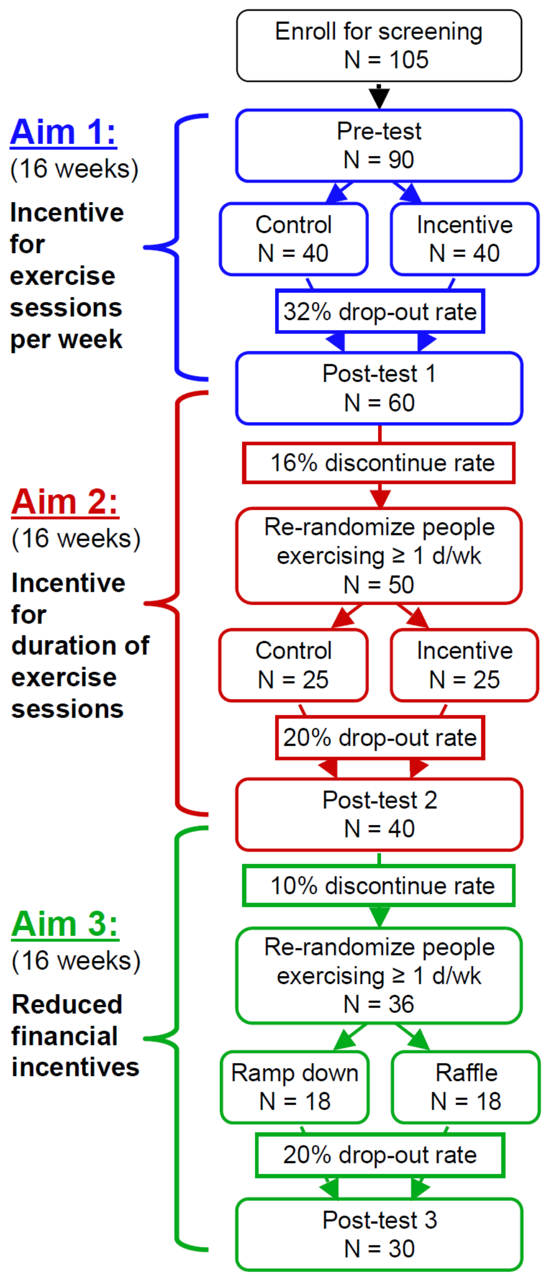

Fig 2. Projected protocol flow.

Protocol: All overweight/obese participants will follow the same protocol. After an initial screening to ensure eligibility, a standard set of clinical outcome assessments will be performed at baseline (Pre-test), after completing 16-weeks of exercise training in Aim 1 (Post-test 1), again after completing the second 16-week exercise period in Aim 2 (Post-test 2), and once more after completing the 16-week exercise period in Aim 3 (Post test 3). Normal weight participants enrolled into Aim 4 will complete only the screening and baseline tests; they will not continue with the incentivized exercise program.

Screening: During this visit, informed written consent/ assent of participants and their parents will be obtained in accordance with IRB guidelines. A medical history and physical exam will be performed. Each child and a parent/guardian will be interviewed to educate them on their child's health status, the importance of physical activity as a component of a healthy lifestyle, and the potential improvements in clinical outcomes that may occur in response to increased exercise behavior. The interview will also be used to assess potential barriers to change. This may include, but is not limited to several identifiable personal and environmental

IRB APPROVED
IRB NUMBER: 0434
IRB APPROVAL DATE: 01/04/2016

concerns that could interfere with the child's ability to complete the study, such as family employment demands, income and education status, transportation and food availability, social structure, and competing time demands. A comprehensive, individualized plan will be developed to reduce barriers. A motivation assessment questionnaire with established validity and reliability (62, 73, 91) and adapted for a rural community will be used to evaluate preferred types of exercise, social support, and perceptions of barriers to exercise by child and family. Community and environmental barriers to exercise will also be assessed. The Choctaw Nation of Oklahoma offers several social service programs designed to eliminate distress, including academic tutoring, mentoring, organized activities, housing assistance, nutrition classes, and counseling. Many of these services are located near the Wellness Centers. The interviewer will be responsible for linking the family to these services when appropriate and performing follow-up to address new and existing barriers. The goal of this effort is to insure that a decision by the child or family to discontinue the study is due to choice and not due to barriers that can be reasonably addressed.

Baseline pre-training testing: The baseline tests may be combined with the screening visit. Baseline tests will be comprised of measurements of daily physical activity, aerobic fitness, body composition, and insulin resistance. Daily physical activity will be objectively measured with a step-activity monitor worn for 7 days during waking hours. Participants will be asked to maintain their normal lifestyle patterns during this time. A registered dietitian will provide instruction on a healthy eating plan but specific weight loss goals or dietary monitoring will not be part of this study. Insulin resistance and other clinical outcomes will be assessed using fasting blood samples. Participants will report to the research center in the morning following an overnight fast. Body composition and exercise tests will be performed as described below.

Exercise training: The 16-week Aim 1 exercise training period will begin within two weeks after the Pre-test. Training sessions will be performed at one of the Wellness Centers described in Facilities, with potential for additional sites depending on needs and the availability of appropriate supervision. All participants already have free membership to the Wellness Centers as a benefit provided to Certificate Degree of Indian Blood (CDIB) cardholders. Free transportation will be arranged through a shuttle service already in place for medical visits. Fitness professionals at each center will provide instruction, supervision, and ongoing guidance to assure that exercise sessions are completed safely and effectively. Participants and their parent/guardian will be instructed that the exercise goal throughout the entire 48-week study is to achieve 60 min/day of moderate-to-vigorous physical activity (MVPA), consistent with current guidelines for children (13, 31, 90). MVPA is defined as continuous exercise that elicits a heart rate ≥ 70% of peak heart rate. Each participant will be able to choose the frequency of visits to the wellness center and the length of time engaged in MVPA, since the purpose of the study is to test the effect of incentives on exercise behavior. Several types of exercise can be selected, including walking, running, cycling, stair stepping, aerobic dance or other activities. Instructors will advise a gradual increase in exercise intensity and duration as tolerated over the first 2-3 weeks to allow for familiarization and safety. To promote healthy behavior within the community and foster social support, all participants will be invited to exercise with family members or friends if they choose and this will be recorded as a covariate. As in our previous studies, exercise intensity will be monitored by recording heart rate with a chest strap transmitter that outputs to a computer for quantification and data storage. These data will be used to document training sessions. To provide backup in the event of technical failure, training sessions will also be recorded in an exercise log by each participant and confirmed by fitness center staff. **Exercise will be quantified by frequency** (sessions per week), **duration** (length of sessions), and **total volume** (Metabolic Equivalent of Task-hours, MET-h, a measure of intensity relative to resting x activity time).

After completing Aim 1 and the associated post-test, all participants who completed an average of ≥ 1 exercise session per week will be eligible to continue on to Aim 2. The only difference between Aims 1 and 2 will be the financial incentive scheme; the overall exercise goals, and provision of transportation and professional oversight will remain the same. Similarly, after completing Aim 2 and the associated post-test, all participants who have averaged ≥ 1 exercise session per week in Aim 2 will be eligible to continue on to Aim 3. No changes will be made to the exercise goals or availability of transportation and facilities in Aim 3.

To allow for potential schedule conflicts and missed sessions (e.g., illness, travel) participants will be allowed to "catch-up" by performing up to 3 extra sessions within the 3 weeks following a week with less than 3 sessions. Thus, if an entire week is missed due to illness, 12 sessions for payment can be completed over the next 3 weeks instead of the targeted 9 sessions. Additionally, participants will not be discouraged from performing more than the targeted exercise volume of 3 days per week of 60 minutes per session. Exercise beyond this target that is performed at school, at home or at the Wellness Centers is acceptable, since the goal

The University of Oklahoma IRB APPROVED AAHRPP
IRB NUMBER: 0434
IRB APPROVAL DATE: 01/04/2016

of this program is to foster increased physical activity. Participants will be instructed to include such extra physical activity in their exercise log book, though they will not receive payment for days or time beyond the structured incentive program. This information will be used as part of the data analysis plan if applicable.

Post-training testing: After each of the 16-week exercise phases in Aims 1, 2, and 3, the clinical outcome tests performed at the baseline will be repeated. Staff that supervise exercise sessions will not be blinded to group assignment, but most other staff, including nurses and chemistry lab staff, will not know group assignments, so the potential for bias is expected to be low. The fasting blood sample will be acquired 2 days (38-48 hours) after the last exercise session in order to assess exercise adaptations that are distinct from the residual effects of the most recent training session. The exercise fitness and body composition tests will also be repeated at the end of each study phase.

Group assignment: Within each Aim, participants will be assigned to one of two groups using a randomized stratified block design that was selected to reduce the potential for baseline variability among the groups. Participants will be assigned to blocks of 2 (duos) matched for age and sex. A participant who is not yet matched on the two factors will determine the age and sex for a newly established duo. The next participant to meet those matching criteria will be assigned to that duo. Each duo will have one participant in each of the intervention groups, but the group to which each participant in a block is assigned will be pre-determined by a unique random permutation.

Aim 1 payments, exercise sessions/week:

	Session of the week			
Group	Day 1	Day 2	Day 3	Max.
Control	$4	$4	$4	$12
Incentive	$4	$10	$16	$30

Aim 2 payments, exercise time/session:

	Time per session			
Group	20 min	40 min	60 min	Max.
Control	$4	$4	$4	$12
Incentive	$4	$7	$10	$30

Fig 3. Aims 1 & 2 incentive structures. Values are payments per visit and weekly maximum.

Incentive programs

Overview and rationale: Each participant will receive $50 for completing the initial screening visit and baseline set of clinical and fitness tests. Normal weight participants in Aim 4 will stop after completing the baseline tests. Overweight/obese participants who continue into the exercise program will also receive and $50 each time they complete the end-of-phase set of clinical and fitness tests (3 Post-tests after each 16-week study phase). These payments are similar to amounts used in our recent and ongoing studies of children that have similar procedures and time requirements. The payment schedules for exercise sessions are detailed in each of the separate Aims sections below. We set the payment levels for Aims 1 and 2 based on our experience with exercise studies for adolescents. Payments were selected to be commensurate with the time commitment of participants, as well as the need to create a distinctly higher payment in the incentivized groups so as to elicit the targeted high adherence. The total time for testing and exercise sessions per participant is ~164 hours over ~50 weeks, not including transportation time to and from the participant's home or school and the wellness and testing sites. Depending on group assignment, the range of total compensation to participants who complete all assigned study visits will be $716-1,292, or ~$4.40-7.90 per hour of involvement. We deemed this to be an appropriate compensation/ incentive without inducing undue influence. Payments for screening and baseline test will be arranged at the time of completion using a reloadable debit card provided at no charge to the participants. Thereafter, payments for completed exercise training sessions and Post-exercise tests will be provided every 2 weeks for as long as the participant remains in the study.

Aim 1: This aim will test the hypothesis that the frequency of exercise sessions at the wellness center will vary with the amount of money offered for the number of sessions per week. Participants will be assigned to one of two groups that only differ in the payments for completing their exercise sessions. For both groups, payment will be provided for the number of sessions performed, up to 3 per week. As shown in **Fig 3**, the Control group can earn up to $12 per week at a flat rate of $4 per session, while those in the Incentive group can earn up to $30 per week through an incentive structure meant to encourage exercise on 3 days per week. Qualifying exercise sessions for both groups must be ≥ 20 minutes/session of MVPA during the first 3 weeks, increasing to ≥ 30 minutes/ session of MVPA in weeks 4-16. To allow for rest breaks, the activity time can be accumulated rather than continuous. The plan for recording sessions and making up missed sessions is

Human Research Participant Protection The University of Oklahoma IRB APPROVED AAHRPP
IRB NUMBER: 0434
IRB APPROVAL DATE: 01/04/2016

outlined in the Exercise Training section. By completing all of the targeted 48 exercise sessions participants can earn a maximum of $192 (Control) or $480 (Incentive) for exercise in this Aim.

Aim 2: This aim will test the hypothesis that exercise time per visit will vary with the amount of money offered for the length of exercise sessions. In this aim, as in Aim 1, the Control group will earn $4 per session for up to 3 sessions per week. The Incentive group can earn up to $30/week, with a payment structure based on the length of time for each exercise session (**Fig 3**). The incentive is meant to encourage at least 60 minutes of MVPA per exercise session, consistent with the daily activity recommendations for youth. As in Aim 1, this study phase is 16 weeks so completion of all 48 targeted exercise sessions will result in payments of $192 (Control) or $480 (Incentive).

Aim 3: This aim will determine if exercise behavior developed during the preceding 32 weeks will continue when payments for exercise are diminished. Participants will be randomized to either the Ramp-down or Raffle group. Maximum payments for the Ramp-down group will begin at $20/week, through a payment structure that incentivizes exercise time, as in Aim 2, with a bonus for completing 3 sessions in a week (**Fig 4**). The weekly payments will be decreased over 8 weeks, reaching $0 for weeks 9-16. The Raffle group will earn chances to win prize money based on the number of fitness center exercise sessions and time spent exercising each week. The number of raffle entries earned for each session will vary from 1-16 per participant each week (**Fig 4**). Random drawings will be conducted to determine winners, with the total value of the weekly prize pool set to ~$7 per person. All participants will continue to receive the same transportation and Wellness Center access as before. Total potential payment for exercise in Aim 3 will be ~$112 per participant, though the actual amount for Raffle group members will vary according to chance.

Clinical outcome tests

Insulin sensitivity: Fasting insulin sensitivity will be assessed by Homeostasis Model of Assessment - Insulin Resistance (HOMA-IR), calculated as: [fasting insulin (μIU/ml) x fasting glucose (mg/dl)/405].

Anthropometry and blood pressure: Height, body mass, and waist and hip circumference will be measured by trained nurses. As regularly performed by our lab, total body and regional fat and lean tissue will be measured using bioelectrical impedance analysis at each of the pre- and post-training visits. Blood pressure will be measured in duplicate after the child has rested quietly for 15 minutes using an appropriately sized arm cuff.

Fitness testing: Bicycle ergometer tests with increasing workloads will be used to measure maximal aerobic work output, peak rate of oxygen consumption (VO_2peak) and heart rate, and the workload corresponding to a heart rate of 170 beats/min (48, 77). Submaximal stages will be used to establish relationships among heart rate and power and used to assess work economy. Continuous measurements of expired gases will be performed with a facemask and metabolic measurement cart and heart rate with Polar heart rate monitors. For normal weight participants, the cut point for fitness level (low versus high) will be a VO_2peak of 46 ml/kg/min for boys and 37 ml/kg/min for girls. This threshold was identified as a marker of elevated metabolic risk, based on a composite of standard clinical outcomes such as blood pressure, glucose, lipids, and waist circumference, in a cohort of more than 2,000 adolescents (age ~15 y), who performed a standard bicycle fitness test (4).

Aim 3 Ramp-down payments:

Week	Time per session 20 min	40 min	60 min	3d/wk Bonus	Max.
1	$2	$4	$6	$2	$20
2	$2	$4	$6	$2	$20
3	$2	$4	$5	$2	$17
4	$2	$4	$4	$2	$14
5	$1	$2	$4	$2	$14
6	$1	$2	$3	$1	$10
7	$1	$1	$3	$1	$10
8	$1	$1	$2	$1	$7

Aim 3 Raffle chances earned:

Exercise time per session	Session of the week Day 1	Day 2	Day 3	Max.
20 min	1	2	3	6
40 min	2	3	5	10
60 min	4	5	7	16

Fig 4. Aim 3 incentive schemes. The Ramp-down bonus is for completing 3 sessions/week. Max. = total payment or raffle chances per week.

Physical activity assessment: Free-living daily ambulatory activity will be measured with accelerometers worn on the waist (Fitbit Zip, Fitbit Inc.) throughout the day, recording data each minute for 7 days. Data analysis includes total step count and activity patterns based on step rates. We have recently completed validation and reliability assessments of

IRB APPROVED
IRB NUMBER: 0434
IRB APPROVAL DATE: 01/04/2016

these monitors in our laboratory have found very high levels of agreement with other waist or ankle-worn accelerometers.

Dietary advice: There will not be a specific dietary intervention in the current investigation. However, all participants will watch a video presentation on healthy eating prepared by a registered dietitian. The dietician will be available for additional consultation if requested by the participant or their family. Participants will be instructed to standardize their food intake for 3 days prior to prior to each fasting blood draw.

Plasma/serum analysis: In addition to measurements of glucose and insulin performed to measure insulin resistance, blood will be collected for measurement of lipids, inflammatory and related disease risk markers. All planned assays are established on campus or in our laboratory. Lipid analyses include total-, HDL- and LDL-cholesterol (measured at the Oklahoma Veterans Administration Clinical Laboratory) and non-esterified fatty acids (colorimetric assay, Wako Chemicals, Richmond, VA). Pro-inflammatory biomarkers associated with obesity and insulin resistance include oxidized LDL (oxLDL) and HDL (oxHDL), high-sensitivity C-reactive protein, myeloperoxidase, high-molecular weight adiponectin, visfatin, and the soluble vascular-derived molecules, intercellular cell adhesion molecule-1 (ICAM-1) and vascular cell adhesion molecule-1 (VCAM-1), measured with ELISAs from Phoenix Pharmaceuticals, R&D Systems and Millipore. Additionally, we will measure the plasma concentration of a panel of amino acids and their metabolites (particularly isoleucine, leucine, valine, and adiptic acid), and a panel of fatty acyl CoA's, that have been reported to be increased in children and adults with diabetes, and have been shown to predict the future onset of diabetes (2, 3, 8, 16, 39, 57, 64, 74, 75, 83, 101, 104, 105). Hemoglobin A1c will be measured to assess recent history of glycemic regulation and diabetes risk (Choctaw Nation Health Clinic). Blood collection, sample analysis and destruction of unused samples will adhere to NIH, IRB and Choctaw Nation of Oklahoma guidelines.

Data analyses: Since group assignments will be balanced for age and sex, the groups are expected to be similar at baseline. Additional comparisons will be performed to identify demographic or other variables on which the groups might differ (e.g., weight loss) that could affect the primary outcomes. Subsequent analyses will adjust for these potentially confounding variables. Regression models, which adjust for modifier (or confounding) variables identified in initial data exploration, will compare between-group differences in the primary outcomes. Since we anticipate that the number of fitness center visits, which is the primary outcome in Aim 1, will follow a Poisson distribution typical of counts, we will explore a multivariable Poisson regression model to analyze that aim. To assess Aim 2, a more conventional regression model will analyze the primary outcome, which is the total time that participants engage in exercise. The effects of exercise training on secondary outcomes will be analyzed similarly, using multiple linear regression. Analysis of Aim 3 will also utilize multivariate modeling to determine which factors, including exercise group assignment, best explain the change in exercise volume and clinical outcomes over the final phase of the study. Residuals generated from regression models will be inspected for normality and data will be log-transformed or subjected to non-parametric testing as appropriate. Aim 4 is a cross-sectional study design so the main effects of physical activity (high versus low), fitness (high versus low) and body size (normal weight versus overweight/obese) on biomarkers of metabolic disease risk will be initially determined using an analysis of variance approach, with Bonferroni tests to make pairwise comparisons between groups. A multivariate modeling approach, similar to that used in Aims 1-3, will be used to identify the best sets of predictor variables for specific biomarkers. A P value <0.05 will be considered statistically significant.

Sample size and power estimates: Group sample sizes were estimated to test the primary hypotheses in Aims 1 and 2 that the Incentive groups will complete a greater number of exercise sessions (Aim 1) and greater total exercise time (Aim 2) than the respective Control groups. For Aim 1, the number of completed exercise sessions is more likely to follow a Poisson distribution than a normal distribution. Accordingly, PASS software (v.2005) was used to explore a Poisson regression model. It was verified that two groups of 40 participants afford 80% power to detect between-group ratios of 1.1 to 1.4 in the number of completed exercise sessions, depending on the groups' mean exercise attendance. For example, if the number of exercise sessions completed by members of the Control group is as low as 15, the study affords 80% power to detect a difference in attendance if the true number of visits in the Incentive group is 1.17 x 15 = 18 visits.

Since few data are available that can be used to estimate rates of participation in incentivized exercise training in this population, we made assumptions based on our collective prior history of exercise and clinical trials. Planned dropout rates were set at ~32% during Aim 1, and ~20% in each of Aims 2 and 3 (**Fig 2**). We reasoned that participants who were the least motivated or who experienced the highest barriers to

The University of Oklahoma IRB APPROVED AAHRPP
IRB NUMBER: 0434
IRB APPROVAL DATE: 01/04/2016

participation were most likely to discontinue during Aim 1 and those that remained would be more likely to complete the entire 48-week study. In Aim 2, we are confident that conventional regression models can be used to analyze total exercise time, but are prepared to transform the outcome if there is evidence that the model's inference might be untrustworthy. Assuming that total exercise time in Aim 2 will be somewhat variable (SD=8 hours), two groups with N = 32 will afford 80% power to detect a between-group difference of 5.75 hours in mean exercise time (out of a total of 48 hours possible). The secondary outcomes in each Aim will focus on changes in insulin sensitivity, body composition, fitness, and the related clinical measurements. The first of the secondary hypotheses tested will be that the Incentive groups in Aims 1 and 2 will have greater changes in clinical outcomes compared to their corresponding Control groups. These comparisons will be performed in separate two-factor analyses of variance where group is a non-repeated effect and time (0, 16, 32 weeks) is a repeated effect. An additional secondary hypothesis, that changes in the secondary outcomes will relate to the volume of exercise participation, will be explored in regression models that pool results from all participants since group assignment per se should not affect the impact if exercise on clinical outcomes.

<u>Expected outcomes, interpretations and potential challenges</u>: The ideal result from this study would be the finding that providing transportation, professional supervision and a financial incentive promotes an increase in exercise frequency and duration in the targeted group. From our past clinical and research involvement we have high confidence that there are an adequate number of eligible youth in the Choctaw Nation Service Area and that initial commitment will be high. The challenge facing all lifestyle intervention programs is adherence, which prompted the approach in this proposal of reinforcing healthy behavior with financial rewards. If incentivized participants meet or exceed the target levels of physical activity, this new strategy for establishing physical activity goals may be useful in other groups with similar barriers. If however, there is unexpectedly low adherence we will carefully explore the reasons for discontinuing exercise, including financial, social, psychological, and temporal, so that the protocol can be revised as needed to limit potential barriers to the greatest extent possible. To facilitate effective adjustments to the approach should it be needed, we will conduct frequent reviews of recruitment and retention benchmarks, perform continuous evaluation of the participant interview results, and convene regular meetings among the project staff.

A variety of referral and advertising strategies will be used to meet enrollment goals. We successfully recruited obese participants of similar age, socio-economic level and health status in the TODAY Study and other protocols, and the PI has regularly provided clinical care to youth at the Choctaw Nation Health Center for the last 11 years. To encourage enrollment and participant compliance and to reduce the amount of travel required for participants, we arranged for free transportation to and from exercise sessions, which can be performed locally at multiple wellness centers in SE Oklahoma. To assure that exercise instruction and supervision is similar among fitness centers we will distribute a manual of standard operating procedures, which will assure safety and fidelity of procedures among sites. Ongoing reviews and revisions will be performed to assure compliance. All clinical tests will be performed at the Choctaw Nation Health Center in Hugo or Talihina, OK to reduce variation in those outcomes. Other challenges will be resolved as they arise.

<u>Limitations and alternate approaches</u>: After careful consideration of the options, we concluded that the proposed study design would be the most effective strategy for addressing our goals in the target population. However, we recognize the possible limitations of this study and have made plans to address the limitations that may arise. **(1)** Targeted weight loss is not included even though obese participants will be enrolled. Weight loss is an important goal for lifestyle intervention programs, but as previously noted, including a weight loss component in the present study design would be harder to implement and monitor successfully and would dilute our ability to test incentives for exercise behavior and evaluate clinical responses to exercise. Some participants may lose weight during the intervention, and we anticipate that changes in body fat may explain part of the variance in changes in insulin sensitivity and other clinical outcomes. This possibility will be addressed as part of the data analysis plan. **(2)** A control group of participants who do not receive compensation for their participation was not included. Traditionally, our research population and IRBs have expected compensation for participation in research, even for control group participants. Thus, all participants will be compensated for exercise behavior, but we believe that the size of the incentives selected will be large enough to elicit a testable difference in behavior between incentivized and control groups. **(3)** A control group of youth who do not exercise was not included. From prior experience and the literature we know that in this population, spontaneous exercise and the likelihood of positive health outcomes without intervention are low, which serves as the impetus for our novel approach of providing a financial incentive and lowering barriers to

IRB APPROVED
IRB NUMBER: 0434
IRB APPROVAL DATE: 01/04/2016

participation. In lieu of a no-exercise group we will acquire an objective, albeit short-term (1 week) measure of daily ambulatory physical activity at baseline, which we anticipate will show the participants as previously sedentary. **(4)** The exercise program in each Aim of the study is 16 weeks long, and significant changes in some clinical outcomes may require more or less time to emerge. This challenge, which exists in nearly all lifestyle intervention studies, requires a balance between the plausible length of time needed for differences to emerge among study groups and the practical challenges of retaining participants in longer interventions or frequent intermediate testing. We reasoned that 16 weeks would be sufficient for changes in insulin sensitivity and body fat to occur if the participants approach or exceed the targeted exercise volume of 3 days per week for 60 minutes. Since the full study duration is 48 weeks, analysis of the changes from baseline to Post tests #2 or #3 can reveal significant changes in clinical outcomes that require 32-48 weeks to become evident. **(5)** The study design has three aims performed consecutively by each participant. As a result of dropouts the characteristics of the participants who complete Aim 3 may differ from the overall group at baseline. Thus, we will test for this possibility as part of the data analysis plan and will carefully interpret the results and their implications with this mind. Factors such as age, sex, location, family and social involvement, financial, medical or other differences will be assessed. An alternate approach of recruiting separate groups so that each aim could be conducted independently was deemed impractical at this time due to the larger number of participants and study resources that would be needed. If, however, dropout rates exceed projections we will reconsider this approach. **(6)** This study targets a specific population of obese youth who are at high risk for developing diabetes, living in a rural area, with low average socioeconomic status, and a unique cultural history as American Indians. Therefore, the outcomes of the study may not be fully generalizable to other groups who do not possess these same characteristics. However, since many people in the United States share at least some of these characteristics, it is reasonable to expect that a major portion of the knowledge gained from this study can be used to improve exercise behavior and to address the health risks of other people living under similar conditions of health distress.

Timeline: The procedures required to complete the study are well established in our research centers. We anticipate that staff training and protocol finalization can be completed within the first two months of the project, and recruitment of participants will begin within that time. Ongoing enrollment and testing will continue through the remaining period of support, with the last of the tests in Aim 3 by the end of year 5. Sample analysis, data processing and preparation of manuscripts presenting the primary outcomes will be ongoing throughout the project. The research team will meet regularly (weekly by phone and monthly in person, or more frequently as needed) to discuss progress and challenges and to review the integrity of the protocol, procedures, and data.

Future plans: Our long-term goal is to identify the types of incentives, barrier reduction, and lifestyle strategies that are most effective at reducing diabetes risk, and which are most likely to persist. If the proposed strategy is found to be effective in increasing exercise participation among youth, we will define optimal types and amounts of incentives in future work. We will also test whether these approaches might be extended more broadly to other adolescent and adult groups with similar characteristics.

Human Research Participant Protection The University of Oklahoma IRB APPROVED AAHRPP
IRB NUMBER: 0434
IRB APPROVAL DATE: 01/04/2016

BIBLIOGRAPHY

1. **Aaron DJ, Kriska AM, Dearwater SR, Cauley JA, Metz KF, and LaPorte RE.** Reproducibility and validity of an epidemiologic questionnaire to assess past year physical activity in adolescents. *Am J Epidemiol* 142: 191-201, 1995.
2. **Adams SH.** Emerging perspectives on essential amino acid metabolism in obesity and the insulin-resistant state. *Adv Nutr* 2: 445-456, 2011.
3. **Adams SH, Hoppel CL, Lok KH, Zhao L, Wong SW, Minkler PE, Hwang DH, Newman JW, and Garvey WT.** Plasma Acylcarnitine Profiles Suggest Incomplete Long-Chain Fatty Acid β-Oxidation and Altered Tricarboxylic Acid Cycle Activity in Type 2 Diabetic African-American Women. *J Nutr* 139: 1073-1081, 2009.
4. **Adegboye ARA, Anderssen SA, Froberg K, Sardinha LB, Heitmann BL, Steene-Johannessen J, Kolle E, and Andersen LB.** Recommended aerobic fitness level for metabolic health in children and adolescents: a study of diagnostic accuracy. *Br Med J* 45: 722-728, 2011.
5. **Ainslie G.** Specious reward: a behavioral theory of impulsiveness and impulse control. *Psychol Bull* 82: 463-496, 1975.
6. **Alessi SM, Badger GJ, and Higgins ST.** An experimental examination of the initial weeks of abstinence in cigarette smokers. *Exp Clin Psychopharmacol* 12: 276-287, 2004.
7. **American College of Sports Medicine.** The recommended quantity and quality of exercise for developing and maintaining cardiorespiratory and muscular fitness, and flexibility in healthy adults. *Med Sci Sports Exerc* 30: 975-991, 1998.
8. **Anderson SG, Dunn WB, Banerjee M, Brown M, Broadhurst DI, Goodacre R, Cooper GJS, Kell DB, and Cruickshank JK.** Evidence that multiple defects in lipid regulation occur before hyperglycemia during the prodrome of type 2 diabetes. *PLoS One* 9: e103217, 2014.
9. **Balagopal P, George D, Patton N, Yarandi H, Roberts WL, Bayne E, and Gidding S.** Lifestyle-only intervention attenuates the inflammatory state associated with obesity - a randomized controlled study in adolescents. *J Pediatr* 146: 342-348, 2005.
10. **Balagopal P, George D, Sweeten S, Mann KJ, Yarandi H, Mauras N, and Vaughan DE.** Response of fractional synthesis rate (FSR) of fibrinogen, concentration of D-dimer and fibrinolytic balance to physical activity-based intervention in obese children. *J Thromb Haemost* 6: 1296-1303, 2008.
11. **Balagopal P, George D, Yarandi H, Funanage V, and Bayne E.** Reversal of obesity-related hypoadiponectinemia by lifestyle intervention: a controlled, randomized study in obese adolescents. *J Clin Endocrinol Metab* 90: 6192-6197, 2005.
12. **Ball GD, Huang TT, Gower BA, Cruz ML, Shaibi GQ, Weigensberg MJ, and Goran MI.** Longitudinal changes in insulin sensitivity, insulin secretion, and beta-cell function during puberty. *J Pediatr* 148: 16-22, 2006.
13. **Barlow SE and the Expert Committee.** Expert committee recommendations regarding the prevention, assessment, and treatment of child and adolescent overweight and obesity: summary report. *Pediatrics* 120: S164-192, 2007.
14. **Basu R, Breda E, Oberg AL, Powell CC, Dalla Man C, Basu A, Vittone JL, Klee GG, Arora P, Jensen MD, Toffolo G, Cobelli C, and Rizza RA.** Mechanisms of the age-associated deterioration in glucose tolerance: contribution of alterations in insulin secretion, action, and clearance. *Diabetes* 52: 1738-1748, 2003.
15. **Bell LM, Watts K, Siafarikas A, Thompson A, Ratnam N, Bulsara M, Finn J, O'Driscoll G, Green DJ, Jones TW, and Davis EA.** Exercise alone reduces insulin resistance in obese children independently of changes in body composition. *J Clin Endocrinol Metab* 92: 4230-4235, 2007.
16. **Berlett BS and Stadtman ER.** Protein oxidation in aging, disease, and oxidative stress. *J Biol Chem* 272: 20313-20316, 1997.
17. **Boule NG, Weisnagel SJ, Lakka TA, Tremblay A, Bergman RN, Rankinen T, Leon AS, Skinner JS, Wilmore JH, Rao DC, and Bouchard C.** Effects of exercise training on glucose homeostasis: The HERITAGE Family Study. *Diabetes Care* 28: 108-114, 2005.
18. **Brigham G, Winhusen T, Lewis D, and Kropp F.** Incentives for retention of pregnant substance users: a secondary analysis. *J Subst Abuse Treat* 38: 90-95, 2010.

Human Research Participant Protection
The University of Oklahoma
IRB APPROVED
AAHRPP
IRB NUMBER: 0434
IRB APPROVAL DATE: 01/04/2016

19. **Brooks GA, Butte NF, Rand WM, Flatt J-P, and Caballero B.** Chronicle of the Institute of Medicine physical activity recommendation: how a physical activity recommendation came to be among dietary recommendations. *Am J Clin Nutr* 79: 921S-930, 2004.
20. **Bush NC, Darnell BE, Oster RA, Goran MI, and Gower BA.** Adiponectin is lower among African Americans and is independently related to insulin sensitivity in children and adolescents. *Diabetes* 54: 2772-2778, 2005.
21. **Casazza K, Willig AL, Gower BA, Nagy TR, Hunter GR, Wallace S, Amaya M, Franklin F, Beasley M, and Fernandez JR.** The role of European genetic admixture in the etiology of the insulin resistance syndrome in children: are the effects mediated by fat accumulation. *J Pediatr* 157: 50-56, 2010.
22. **Centers for Disease Control and Prevention.** County-level estimates of obesity and diabetes among adults in the United States 2007. *MMWR CDC Surveill Summ* 58: 1259-1263, 2007.
23. **Charness G and Gneezy U.** Incentives to exercise. *Econometrica* 77: 909-931, 2009.
24. **Choctaw Nation Health Services.** *State of the Nation's Health 2010: An Addendum Report*. Talihina, OK, 2010.
25. **Church TS, Earnest CP, Skinner JS, and Blair SN.** Effects of different doses of physical activity on cardiorespiratory fitness among sedentary, overweight or obese postmenopausal women with elevated blood pressure: a randomized controlled trial. *JAMA* 297: 2081-2091, 2007.
26. **Church TS, Finley CE, Earnest CP, Kampert JB, Gibbons LW, and Blair SN.** Relative associations of fitness and fatness to fibrinogen, white blood cell count, uric acid and metabolic syndrome. *Int J Obes Relat Metab Disord* 26: 805-813, 2002.
27. **Colberg SR, Sigal RJ, Fernhall B, Regensteiner JG, Blissmer BJ, Rubin RR, Chasan-Taber L, Albright AL, and Braun B.** Exercise and type 2 diabetes. *Diabetes Care* 33: e147-e167, 2010.
28. **Copeland KC, Zeitler P, Geffner ME, Guandalini C, Higgins J, Hirst K, Kaufman FR, Linder B, Marcovina S, McGuigan P, Pyle L, Tamborlane W, and Willi S.** Characteristics of adolescents and youth with recent-onset type 2 diabetes: the TODAY cohort at baseline. *J Clin Endocrinol Metab* Published online Oct 20, 2010.
29. **Cowie CC, Rust KF, Byrd-Holt DD, Eberhardt MS, Flegal KM, Engelgau MM, Saydah SH, Williams DE, Geiss LS, and Gregg EW.** Prevalence of diabetes and impaired fasting glucose in adults in the U.S. populations. *Diabetes Care* 29: 1263-1268, 2006.
30. **Cowie CC, Rust KF, Ford ES, Eberhardt MS, Byrd-Holt DD, Li C, Williams DE, Gregg EW, Bainbridge KE, Saydah SH, and Geiss LS.** Full accounting of diabetes and pre-diabetes in the U.S. Population in 1988-1994 and 2005-2006. *Diabetes Care* 32: 287-294, 2009.
31. **Davis MM, Gance-Cleveland B, Hassink S, Johnson R, Paradis G, and Resnicow K.** Recommendations for prevention of childhood obesity. *Pediatrics* 120: S229-253, 2007.
32. **Diabetes Prevention Program Research Group.** 10-year follow-up of diabetes incidence and weight loss in the Diabetes Prevention Program Outcomes Study. *Lancet* 374: 1677-1686, 2009.
33. **Diabetes Prevention Program Research Group.** Reduction in the incidence of type 2 diabetes with lifestyle intervention or metformin. *N Engl J Med* 346: 393-403, 2002.
34. **Doerfling P, Kopec JA, Liang MH, and Esdaile JM.** The effect of cash lottery on response rates to an online health survey among members of the Canadian Association of Retired Persons: a randomized experiment. *Can J Public Health* 101: 251-254, 2010.
35. **Duncan GE.** Prevalence of diabetes and impaired fasting glucose levels among US adolescents: National Health and Nutrition Examination Survey, 1999-2002. *Arch Pediatr Adolesc Med* 160: 550-552, 2006.
36. **Edwards B.** Childhood obesity: A school-based approach to increase nutritional knowledge and activity levels. *Nurs Clin North Am* 40: 661-669, 2005.
37. **Eisenmann JC, Welk GJ, Wickel EE, and Blair SN.** Stability of variables associated with the metabolic syndrome from adolescence to adulthood: the Aerobics Center Longitudinal Study. *Am J Human Biol* 16: 690-696, 2004.
38. **Eriksson KF and Lindgarde F.** Prevention of type 2 (non-insulin-dependent) diabetes mellitus by diet and physical exercise. The 6-year Malmo feasibility study. *Diabetologia* 34: 891-898, 1991.
39. **Fiehn O, Garvey WT, Newman JW, Lok KH, Hoppel CL, and Adams SH.** Plasma Metabolomic Profiles Reflective of Glucose Homeostasis in Non-Diabetic and Type 2 Diabetic Obese African-American Women. *PLoS ONE* 5: e15234, 2010.
Human Research Participant Protection The University of Oklahoma IRB APPROVED AAHRPP
IRB NUMBER: 0434
IRB APPROVAL DATE: 01/04/2016

40. **Finkelstein EA, Brown DS, Brown DR, and Buchner DM.** A randomized study of financial incentives to increase physical activity among sedentary older adults. *Prev Med* 47: 182-187, 2008.
41. **Finkelstein EA and Kosa KM.** Use of incentives to motivate healthy behaviors among employees. *Gender Issues* 21: 50-89, 2003.
42. **GAO.** Childhood obesity: factors affecting physical activity. Washington, D.C., 2006, p. GAO-07-260R.
43. **Gittelsohn J and Rowan M.** Preventing diabetes and obesity in American Indian communities: the potential of environmental interventions. *Am J Clin Nutr* 93: 1179S-1183S, 2011.
44. **Giuffrida A and Torgerson DJ.** Should we pay the patient? Review of financial incentives to enhance patient compliance. *BMJ* 315: 703-707, 1997.
45. **Goel K, Thomas RJ, Squires RW, Coutinho T, Trejo-Gutierrez JF, Somers VK, Miles JM, and Lopez-Jimenez F.** Combined effect of cardiorespiratory fitness and adiposity on mortality in patients with coronary artery disease. *Am Heart J* 161: 590-597, 2011.
46. **Goldfield GS, Kalakanis LE, Ernst MM, and Epstein LH.** Open-loop feedback to increase physical activity in obese children. *Int J Obes Relat Metab Disord* 24: 888-892, 2000.
47. **Goldfield GS, Mallory R, Parker T, Cunningham T, Legg C, Lumb A, Parker K, Prud'homme D, Gaboury I, and Adamo KB.** Effects of open-loop feedback on physical activity and television viewing in overweight and obese children: a randomized, controlled trial. *Pediatrics* 118: e157-166, 2006.
48. **Gutin B, Yin Z, Humphries MC, Hoffman WH, Gower BA, and Barbeau P.** Relations of fatness and fitness to fasting insulin in black and white adolescents. *J Pediatr* 145: 737-743, 2004.
49. **Harvey-Berino J and Rourke J.** Obesity prevention in preschool Native-American children: A pilot study using home visiting. *Obes Res* 11: 606-611, 2003.
50. **Hu FB, Manson JE, Meir JS, Colditz G, Liu S, Solomon CG, and Willet WC.** Diet, lifestyle, and the risk of Type 2 diabetes mellitus in women. *N Engl J Med* 345: 790-797, 2001.
51. **Iozzo P.** Where does insulin resistance start? The adipose tissue. *Diabetes Care* 32: S168-S173, 2009.
52. **Jeffery RW, Wing RR, Thorson C, and Burton LR.** Use of personal trainers and financial incentives to increase exercise in a behavioral weight-loss program. *J Consult Clin Psych* 66: 777-783, 1998.
53. **John L, Loewenstein G, Troxel A, Norton L, Fassbender J, and Volpp K.** Financial incentives for extended weight loss: a randomized, controlled trial. *J Gen Intern Med* Epub ahead of print Jan 20, 2011, 2011.
54. **Kahneman D and Tversky A.** Prospect theory: an analysis of decision under risk. *Econometrica* 47: 263-292, 1979.
55. **Kelly LA, Lane CJ, Weigensberg MJ, Toledo-Corral CM, and Goran MI.** Pubertal changes of insulin sensitivity, acute insulin response, and B-cell function in overweight Latino youth. *J Pediatr* Published online Sep 30, 2010.
56. **Khort WM, Kirwin JP, Staten MA, Bourey RE, King DS, and Holloszy JO.** Insulin resistance in aging is related to abdominal obesity. *Diabetes* 42: 273-281, 1993.
57. **Kim JY, Park JY, Kim OY, Ham BM, Kim H-J, Kwon DY, Jang Y, and Lee JH.** Metabolic Profiling of Plasma in Overweight/Obese and Lean Men using Ultra Performance Liquid Chromatography and Q-TOF Mass Spectrometry (UPLC−Q-TOF MS). *J Proteome Res* 9: 4368-4375, 2010.
58. **Laaksonen DE, Lindstrom J, Lakka TA, Eriksson JG, Niskanen L, Wikstrom K, Aunola S, Keinanen-Kiukaanniemi S, Laakso M, Valle TT, Ilanne-Parikka P, Louheranta A, Hamalainen H, Rastas M, Salminen V, Cepaitis Z, Hakumaki M, Kaikkonen H, Harkonen P, Sundvall J, Tuomilehto J, Uusitupa M, and for the Finnish Diabetes Prevention Study Group.** Physical activity in the prevention of type 2 diabetes: the Finnish Diabetes Prevention Study. *Diabetes* 54: 158-165, 2005.
59. **Lee S, Bacha F, Gungor N, and Arslanian S.** Comparison of different definitions of pediatric metabolic syndrome: relation to abdominal adiposity, insulin resistance, adiponectin, and inflammatory biomarkers. *J Pediatr* 152: 177-184.e173, 2008.
60. **Lee S, Kuk JL, Katzmarzyk PT, Blair SN, Church TS, and Ross R.** Cardiorespiratory fitness attenuates metabolic risk independent of abdominal subcutaneous and visceral fat in men. *Diabetes Care* 28: 895-901, 2005.
61. **Li C, Ford ES, Zhao G, and Mokdad AH.** Prevalence of pre-diabetes and Its association with clustering of cardiometabolic risk factors and hyperinsulinemia among U.S. adolescents. *Diabetes Care* 32: 342-347, 2009.
Human Research Participant Protection The University of Oklahoma IRB APPROVED AAHRPP
IRB NUMBER: 0434
IRB APPROVAL DATE: 01/04/2016

62. **Litt DM, Iannotti RJ, and Wang J.** Motivations for adolescent physical activity. *J Phys Act Health* 8: 220-226, 2011.
63. **Liu LL, Lawrence JM, Davis C, Liese AD, Pettitt DJ, Pihoker C, Dabelea D, Hamman R, Waitzfelder B, and Kahn HS.** Prevalence of overweight and obesity in youth with diabetes in USA: the SEARCH for Diabetes in Youth Study. *Pediatr Diab* 32: S133-S140, 2009.
64. **Mihalik SJ, Goodpaster BH, Kelley DE, Chace DH, Vockley J, Toledo FGS, and DeLany JP.** Increased Levels of Plasma Acylcarnitines in Obesity and Type 2 Diabetes and Identification of a Marker of Glucolipotoxicity. *Obesity* 18: 1695-1700, 2010.
65. **Moore E, Copeland KC, Parker D, Burgin C, and Blackett PR.** Ethnic differences in fasting glucose, insulin resistance and lipid profiles in obese adolescents. *J Okla State Med Assoc* 99: 439-443, 2006.
66. **Moran A, Jacobs DR, Steinberger J, Hong CP, Prineas R, Luepker R, and Sinaiko AR.** Insulin resistance during puberty: results from clamp studies in 357 children. *Diabetes* 48: 2039-2044, 1999.
67. **Must A, Jacques PF, Dallal GE, Bajema CJ, and Dietz WH.** Long-term morbidity and mortality of overweight adolescents. A follow-up of the Harvard Growth Study of 1922 to 1935. *N Engl J Med* 327: 1350-1355, 1992.
68. **Nassis GP, Papantakou K, Skenderi K, Triandafillopoulou M, Kavouras SA, Yannakoulia M, Chrousos GP, and Sidossis LS.** Aerobic exercise training improves insulin sensitivity without changes in body weight, body fat, adiponectin, and inflammatory markers in overweight and obese girls *Metabolism* 54: 1472-1479, 2005.
69. **Ogden CL, Carroll MD, Curtain LR, McDowell MA, Tabak CJ, and Flegal KM.** Prevalence of overweight and obesity in the United States, 1999-2004. *JAMA* 295: 1549-1555, 2006.
70. **Ogden CL, Carroll MD, and Flegal KM.** High body mass index for age among US children and adolescents, 2003-2006. *JAMA* 299: 2401-2405, 2008.
71. **Oklahoma Department of Public Health.** http://www.ok.gov/health/Child and Family Health.
72. **Pate RR, Pratt M, Blair SN, Haskell WL, Macera CA, Bouchard C, Buchner D, Ettinger W, Heath GW, and King AC.** Physical activity and public health: a recommendation from the Centers of Disease Control and Prevention and the American College of Sports Medicine. *JAMA* 273: 402-407, 1995.
73. **Perry C and Hoffman B.** Assessing tribal youth physical activity and programming using a community-based participatory research approach. *Public Health Nurs* 27: 104-114, 2010.
74. **Randle PJ, Kerbey AL, and Espinal J.** Mechanisms decreasing glucose oxidation in diabetes and starvation: role of lipid fuels and hormones. *Diabetes Metab Rev* 4: 623-638, 1988.
75. **Rao RK and Clayton LW.** Regulation of protein phosphatase 2A by hydrogen peroxide and glutathionylation. *Biochem Biophys Res Commun* 293: 610-616, 2002.
76. **Read D, Loewenstein G, and Rabin M.** Choice bracketing. *J Risk Uncertainty* 19: 171-197, 1999.
77. **Riddoch C, Edwards D, and Page A.** The European Youth Heart Study: cardiovascular disease risk factors in children: rationale, aims, study design, and validation of methods. *J Phys Activ Health* 2: 115-129, 2005.
78. **Roemmich JN, Gurgol CM, and Epstein LH.** Open-loop feedback increases physical activity of youth. *Med Sci Sports Exerc* 36: 668-673, 2004.
79. **Savoye M, Shaw M, Dziura J, Tamborlane WV, Rose P, Guandalini C, Goldberg-Gell R, Burgert TS, Cali AMG, Weiss R, and Caprio S.** Effects of a weight management program on body composition and metabolic parameters in overweight children: a randomized controlled trial. *JAMA* 297: 2697-2704, 2007.
80. **Scott D and Goldwater BC.** Increasing attendance at sport training sessions. *Percept Mot Skills* 87: 515-518, 1998.
81. **Seal KH, Kral AH, Lorvick J, McNees A, Gee L, and Edlin BR.** A randomized controlled trial of monetary incentives vs. outreach to enhance adherence to the hepatitis B vaccine series among injection drug users. *Drug Alcohol Depend* 20: 127-131, 2003.
82. **SEARCH for Diabetes in Youth Study Group, Liese AD, D'Agostino RB, Hamman RF, Kilgo PD, Lawrence JM, Liu LL, Loots B, Linder B, Marcovina S, Rodriguez B, Standiford D, and Williams DE.** The burden of diabetes mellitus among US youth: prevalence estimates from the SEARCH for Diabetes in Youth Study. *Pediatrics* 118: 1510-1518, 2006.
83. **Shah SH, Sun J-L, Stevens RD, Bain JR, Muehlbauer MJ, Pieper KS, Haynes C, Hauser ER, Kraus WE, Granger CB, Newgard CB, Califf RM, and Newby LK.** Baseline metabolomic profiles predict
IRB APPROVED
IRB NUMBER: 0434
IRB APPROVAL DATE: 01/04/2016

cardiovascular events in patients at risk for coronary artery disease. *Am Heart J* 163: 844-850.e841, 2012.
84. **Shaw M, Savoye M, Cali A, Dziura J, Tamborlane WV, and Caprio S.** Effect of a successful intensive lifestyle program on insulin sensitivity and glucose tolerance in obese youth. *Diabetes Care* 32: 45-47, 2009.
85. **Short KR, Vittone JL, Bigelow ML, Proctor DN, Rizza RR, Coenen-Schimke JM, and Nair KS.** Impact of aerobic training on age-related changes in insulin action and muscle oxidative capacity. *Diabetes* 52: 1888-1896, 2003.
86. **Sigal RJ, Kenny GP, Wasserman DH, Castaneda-Sceppa C, and White RD.** Physical activity/exercise and type 2 diabetes. A consensus statement from the American Diabetes Association. *Diabetes Care* 29: 1433-1438, 2006.
87. **Sinha R, Fisch G, Teague B, Tamborlane WV, Banyas B, Allen K, Savoye M, Rieger V, Taksali S, Barbetta G, Sherwin RS, and Caprio S.** Prevalence of impaired glucose tolerance among children and adolescents with marked obesity. *N Engl J Med* 346: 802-810, 2002.
88. **Songer T, Glazner J, Coombs LP, Cuttler L, Daniel M, Estrada S, Klingensmith G, Kriska A, Laffel L, Zhang P, and the TODAY Study Group.** Examining the economic costs related to lifestyle and pharmacological interventions in youth with Type 2 diabetes. *Expert Rev Pharmacoecon Outcomes Res* 1: 315-324, 2006.
89. **Srinivasan SR, Meyers L, and Berenson GS.** Predictability of childhood adiposity and insulin for developing insulin resistance syndrome (syndrome X) in young adulthood: the Bogalusa Heart Study. *Diabetes* 51: 204-209, 2002.
90. **Steinberger J, Daniels SR, Eckel RH, Hayman L, Lustig RH, McCrindle B, and Mietus-Snyder ML.** Progress and challenges in metabolic syndrome in children and adolescents: a scientific statement from the American Heart Association Atherosclerosis, Hypertension, and Obesity in the Young Committee of the Council on Cardiovascular Disease in the Young; Council on Cardiovascular Nursing; and Council on Nutrition, Physical Activity, and Metabolism. *Circulation* 119: 628-647, 2009.
91. **Stevens J, Cornell CE, Story M, S.A. F, Levin S, Becenti A, Gittelsohn J, Going SB, and Reid R.** Development of a questionnaire to assess knowledge, attitudes, and behaviors in American Indian children. *Am J Clin Nutr* 69: 773S-781S, 1999.
92. **Stitzer ML, Polk T, Bowles S, and Kosten T.** Drug users' adherence to a 6-month vaccination protocol: effects of motivational incentives. *Drug Alcohol Depend* 107: 76-79, 2010.
93. **Story M, Evans M, Fabsitz RR, Clay TE, Holy Rock B, and Broussard B.** The epidemic of obesity in American Indian communities and the need for childhood obesity-prevention programs. *Am J Clin Nutr* 69: 747S-754S, 1999.
94. **Story M, Stevens J, and Himes J.** Obesity in American-Indian children: Prevalence, consequences, and prevention. *Prev Med* 37: S3-12, 2003.
95. **Sutherland K, Christianson JB, and Leatherman S.** Impact of targeted financial incentives on personal health behavior. *Med Care Res Rev* 65: 36S-78S, 2008.
96. **Syrenicz A, Garanty-Bogacka B, Syrenicz M, Gebala A, and Walczak M.** Low-grade systemic inflammation and the risk of type 2 diabetes in obese children and adolescents. *Neuro Endocrinol Lett* 27: 435-438, 2006.
97. **Temelkova-Kurktshiev T, Siegert G, Bergmann S, Henkel E, Koehler C, Jaross W, and Hanefeld M.** Subclinical inflammation is strongly related to insulin resistance but not to impaired insulin secretion in a high risk population for diabetes. *Metabolism* 51: 743-749, 2002.
98. **The TODAY Study Group, Zeitler P, Epstein L, Grey M, Hirst K, Kaufman F, Tamborlane WV, and Wilfley D.** Treatment options for type 2 diabetes in adolescents and youth: a study of the comparative efficacy of metformin alone or in combination with rosiglitazone or lifestyle intervention in adolescents with type 2 diabetes. *Pediatr Diab* 8: 74-87, 2007.
99. **Tuomilehto J, Lindstrom J, Eriksson JG, Valle TT, Hamalainen H, Ilanne-Parikka P, Keinanen-Kiukaanniemi S, Laakso M, Louheranta A, Rastas M, Salminen V, and Uusitupa M.** Prevention of Type 2 diabetes mellitus by changes in lifestyle among subjects with impaired glucose tolerance. *N Engl J Med* 344: 1343-1350, 2001.
100. **U.S. Preventive Services Task Force.** Screening for obesity in children and adolescents: US Preventive Services Task Force recommendation statement. *Pediatrics*: 2009-2037, 2010.
IRB APPROVED
IRB NUMBER: 0434
IRB APPROVAL DATE: 01/04/2016

101. **Ussher JR, Koves TR, Jaswal JS, Zhang L, Ilkayeva O, Dyck JR, Muoio DM, and Lopaschuk GD.** Insulin-stimulated cardiac glucose oxidation is increased in high-fat diet-induced obese mice lacking malonyl CoA decarboxylase. *Diabetes* 58: 1766-1775, 2009.
102. **van der Heijden G-J, Toffolo G, Manesso E, Sauer PJJ, and Sunehag AL.** Aerobic exercise increases peripheral and hepatic insulin sensitivity in sedentary adolescents. *J Clin Endocrinol Metab* 94: 4292-4299, 2009.
103. **Volpp KG, John LK, Troxel AB, Norton L, Fassbender J, and Loewenstein G.** Financial incentive-based approaches for weight loss: a randomized trial. *JAMA* 300: 2631-2637, 2008.
104. **Wang TJ, Larson MG, Vasan RS, Cheng S, Rhee EP, McCabe E, Lewis GD, Fox CS, Jacques PF, Fernandez C, O'Donnell CJ, Carr SA, Mootha VK, Florez JC, Souza A, Melander O, Clish CB, and Gerszten RE.** Metabolite profiles and the risk of developing diabetes. *Nat Med* 17: 448-453, 2011.
105. **Wang TJ, Ngo D, Psychogios N, Dejam A, Larson MG, Vasan RS, Ghorbani A, x, Sullivan J, Cheng S, Rhee EP, Sinha S, McCabe E, Fox CS, x, Donnell CJ, Ho JE, Florez JC, Magnusson M, Pierce KA, Souza AL, Yu Y, Carter C, Light PE, Melander O, Clish CB, and Gerszten RE.** 2-Aminoadipic acid is a biomarker for diabetes risk. *J Clin Invest* 123: 4309-4317, 2013.
106. **Weston AT, Petosa R, and Pate RR.** Validation of an instrument for measurement of physical activity in youth. *Med Sci Sports Exerc* 29: 138-143, 1997.
107. **White NH, Pyle L, Tamborlane WV, Geffner ME, Guandalini C, and the TODAY Study Group.** Clinical characteristics and co-morbidities in a large cohort of youth with type 2 diabetes mellitus (T2DM) screened for the Treatment Options for Type 2 Diabetes in Adolescents and Youth (TODAY) Study. *Diabetes* 58(Suppl 1): A70, 2009.
108. **Williams DE, Caldwell BL, Cheng YJ, Cowie CC, Gregg EW, Geiss LS, Engelgau MM, Narayan KM, and Imperatore G.** Prevalence of impaired fasting glucose and its relationship with cardiovascular disease risk factors in US adolescents, 1999-2000. *Pediatrics* 116: 1122-1126, 2005.
109. **Winer JC, Zern TL, Taksali SE, Dziura J, Cali AM, Wollschlager M, Seyal AA, Weiss R, Burgert TS, and Caprio S.** Adiponectin in childhood and adolescent obesity and its association with inflammatory markers and components of the metabolic syndrome. *J Clin Endocrinol Metab* 91: 4415-4423, 2006.
110. **Yeckel CW, Taksali S, Dziura J, Weiss R, Burgert TS, Sherwin RS, Tamborlane WV, and Caprio S.** The normal glucose tolerance continuum in obese youth: evidence for impairment in beta-cell function independent of insulin resistance. *J Clin Endocrinol Metab* 90: 747-754, 2005.
111. **Yeckel CW, Weiss R, Dziura J, Taksali S, Dufour S, Burgert TS, Tamborlane WV, and Caprio S.** Validation of insulin sensitivity indices from oral glucose tolerance test parameters in obese children and adolescents. *J Clin Endocrinol Metab* 89: 1096-1101, 2004.

IRB APPROVED
IRB NUMBER: 0434
IRB APPROVAL DATE: 01/04/2016

PROTECTION OF HUMAN SUBJECTS

1a. Human Subjects Involvement and Characteristics

Proposed involvement: This proposal is a clinical trial to compare how distinct financial incentive programs elicit different volumes of exercise training, and how the volume of exercise ultimately affects insulin sensitivity and clinical outcomes in overweight/obese youth ages 11-21 years old. Within Aims 1-3, children will be assigned to a specific incentive program using a randomized, stratified design, balancing for age and sex. The participants will complete the primary testing procedures before and after each of the 16-week exercise phases that comprise Aims 1-3. For Aim 4, we propose a cross-sectional comparison between the overweight/obese children in Aims 1-3 and comparison groups of normal weight children that are classified as either low or high physical fitness and physical activity. All tests and procedures to be performed have been approved by our institution for use in children in our prior and ongoing studies.

Characteristics of the subject population: Boys and girls between 11 and 21 years of age, who meet the eligibility requirements of the study and are eligible to receive medical services from the Choctaw Nation Health Services (including living in the 10.5 country service area) will be enrolled. Target enrollment for the initial screening visit is 105 overweight or obese youth, with the goal of 80-90 participants completing the baseline testing (this allows for a small number of participants to provide initial consent but then decline participation or be excluded for one or more of the reasons outlined in the Inclusion/Exclusion sections below). Overweight will be defined as ≥ 85th percentile for BMI for age and sex, according to the Center for Disease Control's growth charts. Obesity is defined as ≥ 95th percentile for BMI on the same charts. For normal weight groups, target enrollment will be 40 with low fitness/activity and 40 with high fitness/activity. The cut point for fitness level (low versus high) will be a VO_2peak on the bicycle fitness test of 46 ml/kg/min for boys and 37 ml/kg/min for girls. In a cohort of more than 2,000 adolescents (age ~15 y) who performed a standard bicycle fitness test, participants who were below the cited thresholds displayed elevated metabolic risk, based on a composite of standard clinical outcomes, including blood pressure, glucose, lipids, and waist circumference) (4).

Inclusion criteria: In addition to the age and BMI criteria noted above, overweight/obese youth must have a positive family history of type 2 diabetes (first or second degree relative), and must have reached a maturation level of Tanner stage ≥ 2 breasts (girls) or genitalia (boys). For normal weight participants, diabetes family history will be recorded but will not be a specific inclusion or exclusion criterion. The pubertal staging criteria is included to control for the reported decline in insulin sensitivity that occurs during the transition from Tanner stage 1 to Tanner stage 2 in both boys and girls (12, 21, 55, 66). To our knowledge, there are no studies that have directly reported whether or not Tanner stage affects the responsiveness of insulin sensitivity or other clinical outcomes to interventions such as exercise. A recently published comment and reply with exploratory analyses (105) from a completed study suggested that Tanner stage had little or no effect on how insulin sensitivity responded to an exercise and weight loss intervention for obese adolescents In this proposal we will have the opportunity to address this potentially important question. Pubertal staging will be assessed by a physician or nurse using a visual aid that was originally developed as a self-report questionnaire (106). During the routine physical exam the clinician will assess breast development in girls and pubic hair development in boys and girls and will record the developmental stage.

Exclusion criteria: Screening prior to enrollment will include medical history, physical exam, and blood pressure. Overweight/obese participants will be excluded if they report exercising consistently for more than 3 times per week for more than 30 minutes during the past three months. Physical activity will be assessed with questionnaires validated for use in youth (1, 106) and objectively measured with step activity monitors for 7 days prior to the baseline testing session. Youth will also be excluded before enrollment or during study participation if they have medical conditions, use medications, or have lifestyle features that are known to impact metabolic or physical function in a way that would pose risks or obscure the interpretation of results. Examples include polycystic ovary syndrome, all forms of diabetes (including type 1, type 2, MODY, etc., and defined as HbA1c > 6.5% and/or elevated fasting glucose or 2-hour glucose following a standard 75-gram oral glucose tolerance test within past 6 months), active cardiovascular, endocrine or liver disease, kidney or other organ dysfunction, chronic debilitating disease or impaired physical mobility, anemia, symptoms of

Human Research Participant Protection
The University of Oklahoma
IRB APPROVED
AAHRPP
IRB NUMBER: 0434
IRB APPROVAL DATE: 01/04/2016

undiagnosed illness, history of alcoholism (alcohol use > 4oz/day), tobacco use (regular use within the past 6 months), or substance abuse. All pregnant females or those intending to become pregnant during the study period will also be excluded. Medications such as beta-blockers, metformin, thiazolinediones, corticosteroids, and anticoagulants will be exclusion factors. This is not an exhaustive list of medications and conditions that will be considered as exclusionary. Clinical staff will evaluate each participant to determine if there are conditions or medications that that could either interfere with the scientific goals of the study or pose a risk to participant safety and will exclude participants in such circumstances.

Rationale for involvement of special or vulnerable classes of subjects: No special classes of subjects besides adolescent youth will be included. The rationale for including youth in this proposal is to address the growing problem of sedentary behavior and obesity and the accompanying risk for metabolic and vascular disorders in children. Prospective studies designed to prevent, treat, or reverse the effects of these disorders are urgently needed. The number of children required to reach statistically and clinically meaningful outcomes was estimated from published data acquired from prior studies both in adults and children. Continuous evaluation and refinement of methods and outcomes will be performed so that current and future studies can be rationally designed with the highest scientific value and participant safety in mind, while appropriately managing the number of children involved in the research.

Collaborating sites: Recruiting, screening and outcomes testing will be performed primarily at the Choctaw Nation Health Care Center (Talihina, OK) or the Wellness Center in Hugo, OK in collaboration with faculty and staff from the University of Oklahoma Health Sciences Center (Oklahoma City, OK). Exercise training sessions will occur at multiple fitness facilities operated by the Choctaw Nation in Southeast Oklahoma so that participants will have access to sites nearest to their home. Standard Operating Procedures will be developed and implemented at all fitness centers to assure all participants are treated consistently among all sites. Since the research team has the required resources for recruiting, screening, exercise and outcomes testing at OUHSC, some of these activities can be performed in Oklahoma City as needed to facilitate study progress and accommodate participant availability.

1b. Sources of Materials
Research materials obtained: The primary research samples obtained will include exercise records (comprised of attendance and heart rate data) and clinical outcome tests. Clinical measures will include body composition by bioelectrical impedance analysis, anthropometric values, blood pressure, exercise fitness testing, and supporting data acquired from the medical record and interview (e.g., age, height, weight, race, family history of disease). Additionally, blood samples will be collected for measurements of glucose and insulin (used to measure insulin sensitivity), lipids, inflammatory and oxidative stress markers, and related biomarkers of diabetes risk that have been studied in adults and children.

Access: Only IRB-approved members of the study team will have access to individually identifiable private information. All study records will be kept in locked file cabinets and within secure electronic data storage. All hospital and university employees are required to complete annual HIPPA training. Data or blood samples that are shared with people outside of the study team will be coded and stripped of personal identifying details.

How materials will be obtained: All data, test results and biological samples (blood) obtained during the course of the study will be used exclusively for research purposes. No use will be made of pre-existing specimens.

1c. Potential Risks

1. Blood will be withdrawn for screening as needed and on pre- and post-exercise program visits (approximately 4 months apart). Approximately 10ml of whole blood will be collected. Therefore the total blood withdrawn will not exceed 3ml/kg body mass on a single day or 7 ml/kg body mass over any 6-week period, in accordance with NIH guidelines.
2. Exercise testing, including tests of maximal power output will be performed. The risks associated with maximal exercise testing may include shortness of breath, fatigue, and local muscle discomfort. A heart attack can occur in people who have heart disease. The test will be discontinued if any unusual

The University of Oklahoma IRB APPROVED AAHRPP
IRB NUMBER: 0434
IRB APPROVAL DATE: 01/04/2016

or unexpected pain, discomfort or elevations in blood pressure occur. Emergency measures are immediately available in case of cardiac emergency, although such emergencies are rare (1 incident per every 887,000 hours of exercise).

2. Adequacy of Protection Against Risks

2a. Recruitment and Informed Consent

Plans for recruitment: Volunteers will be recruited through the Choctaw Nation Health Care Center, including the clinics for obesity, adolescent medicine and general pediatrics. Recruitment will also utilize physician referrals, media flyers, electronic bulletin boards, and news media advertisements, all of which will receive approval from the OUHSC and Choctaw Nation IRB before implementation. Our recruitment needs are 105 overweight/obese participants enrolled over the first 4 years of the grant (~20 subjects per year), and up to 80 normal weight participants, which we deem to be feasible based on the population and resources available.

Consent process: Prior to visiting the clinical research center, each participant's parent or legal guardian will receive a detailed description of the study goals, procedures, and expectations. A phone or in-person interview will be used to address initial questions and to collect general demographic data to assure the child and their family will be eligible and willing to participate. Once this step is complete, the volunteers will be scheduled for an initial screening visit at the research center. This visit will begin with the completion of the consent/assent documents. Volunteers and their parents or legal guardians will meet one of the IRB-approved members of the investigative team who will explain the scientific rationale of the study, the procedures, potential risks involved, and rights of the participant. Written consent/assent from the child and consent of their parent/guardian will be obtained using IRB-approved forms. It will be made clear to potential subjects that participation in the study is entirely voluntary and they have the right to discontinue the study at any time without prejudice. A record of consent and participation will be kept at the research center, with copies of the consent given to the participant and the original kept with the investigator's study records.

2b. Protections Against Risk

The Choctaw Nation of Oklahoma and OUHSC Institutional Review Boards will approve all protocols and procedures prior to initiation of the studies. Participants will be provided with 24-hour phone numbers and email addresses for the PI or other study personnel if they have questions or concerns regarding their participation in the study. They may also contact the IRB with problems or concerns. The following protection will be taken for the risks identified above:

1) The amount of blood drawn is within the amounts acceptable by the institution and NIH. The amount of blood drawn will be recorded. Since only 10ml of whole blood will be collected every 16 weeks there is minimal risk for participants to donate blood during the study, although they will be requested not to do so.
2) A Data Safety Monitoring Plan (DSMP) will be utilized for patient safety. A Data Safety Monitoring Board (DSMB) is not necessary for this proposal because it is a non-blinded study conducted at a single site. The Investigative team are experienced in all procedures in the study. The investigative team will review all participant safety issues as delineated in the DSMP.
3) Confidentiality of all medical records will be strictly maintained by established procedures. The original study data will be kept in the study facility and entered into a secure computer database. Physical records will be stored in locked cabinets and electronic records through security pass words. The principal investigator reviews all data. Violations of confidentially will be immediately reported to the IRB. Study records will not identify subjects by name but using a numeric code. These systems are currently in place to protect against loss of confidentiality and undergo periodic revision and improvement according to university guidelines.

3. Potential Benefits of the Proposed Research to Human Subjects and Others

Each participant will undergo a standard physical exam, medical history, fitness assessment, body composition testing, and measurement of several clinically important blood components (e.g., glucose, insulin, lipids). This information will be provided to the participants and their parents and could give them beneficial insight into their health. All test results will be reviewed by the study team, including a qualified physician, and participants will be promptly informed of any outcomes for which they should consider seeking additional medical testing and advice. Overweight/obese participants will be enrolled in a supervised exercise program. In our hands such

The University of Oklahoma IRB APPROVED AAHRPP
IRB NUMBER: 0434
IRB APPROVAL DATE: 01/04/2016

programs are safe and effective at improving fitness and health. In this environment they will have the opportunity to interact with other participants in the exercise center and with health and fitness experts. Participants and their families may also appreciate that they are contributing to the advancement of medical science. In our opinion, the potential for beneficial changes in health and fitness resulting from exercise training outweigh the potential minimal risks arising from the study procedures.

4. Importance of the Knowledge to be Gained

Adolescent children who are obese and sedentary have increased risk to develop metabolic and cardiovascular disorders. Regular exercise may help positively modify the level of risk but not enough is known about how to elicit effective exercise participation. This proposal will determine how variation of financial incentives alters exercise participation and how the resulting volume of exercise affects metabolic health in previously sedentary obese youth. Participation in a supervised exercise program is expected to provide at least minimal to moderate benefit in this population, as assessed by changes in insulin sensitivity, cardiorespiratory fitness, body composition, lipids, and inflammatory markers. The present study will also contribute directly to the goal of developing rational and effective strategies for preventing or reversing trends in obesity, diabetes, and cardiometabolic risk. In our opinion, these benefits outweigh the minimal risks posed to the human volunteers involved in the proposed studies.

5. Data and Safety Monitoring Plan

A Data Safety Monitoring Plan (DSMP) will be utilized for patient safety. A Data Safety Monitoring Board (DSMB) is not necessary for this proposal because it is a non-blinded study conducted at a single site. The Investigative team are experienced in all procedures in the study. The investigative team will review all participant safety issues as delineated in the DSMP.

5a. Monitoring and Reporting of Unexpected Adverse Events

The procedures in this study pose minimal risk and the number and severity of unexpected adverse events is low. The primary sources of unexpected events are from placing intravenous catheters and exercise. The risks associated with phlebotomy procedures include local bruising, bleeding, temporary color changes of the skin as well as a slight chance of infection at the site of the needle stick, and becoming lightheaded and fainting during the procedure. During exercise there is risk of falls or other form of injury, fatigue, nausea, muscle soreness, or myocardial infarction in susceptible individuals. We will monitor for these adverse events by obtaining vital signs on each study test visit as well as observing and communicating with subjects throughout the testing procedures.

Dr. Kenneth Copeland, MD, a pediatric endocrinologist and primary investigator for the study, will provide medical oversight. If unexpected events occur, the Principle Investigator will advise the IRB. Unanticipated non-serious adverse events will be reported promptly according to Choctaw Nation of Oklahoma and OUHSC IRB guidelines. Serious adverse events will be reported within 24 to 48 hours. Adverse events will be recorded and classified using the following grading and attribution scale:

Grading = Mild, Moderate, Severe
Attribution = Definite, Probable, Possible, Unlikely, Unrelated

In the absence of moderate or serious adverse events, as is appropriate with minimal risk studies, reports of study progress will be submitted to the IRB for review on an annual basis or at the end of the study, whichever comes first.

5b. Termination Criteria

Criteria for early withdrawal of individuals from this study are: the occurrence of a serious adverse event, or a participant's inability or unwillingness to adhere to study procedures. Criteria for early termination of the study include an unexpectedly high rate of serious adverse events that significantly increase the risk-to-benefit ratio of study participation as evaluated by the IRB.

Participants may leave, or be required to leave the study for the following reasons:

1) Request by the subject to leave the study

IRB APPROVED
IRB NUMBER: 0434
IRB APPROVAL DATE: 01/04/2016

2) Evidence of deliberate non-compliance
3) Pregnancy
4) Alcohol abuse; illicit drug abuse
5) Development of chronic conditions that are likely to impact metabolic variables (either directly or due to required medications), or result in a participant being unable to complete the required tests. Such conditions could include, but are not limited to, diabetes, hypothyroidism or other endocrine disorders, rheumatoid arthritis requiring steroids or limiting mobility, cardiovascular disease, stroke, or cardiac failure, neurological disorders such as multiple sclerosis or cancer, or other organ disorders.
6) Development of acute conditions that are likely to impact metabolic and vascular outcomes (either directly or due to required medications), or result in the subject being unable to participate; e.g. motor vehicle accident with multiple fractures, myocardial infarction, major depression.

This does not represent a comprehensive listing of criteria or causes. The investigators will report early participant withdrawal and the reason for withdrawal as part of regular safety monitoring procedures as outlined by the IRB.

6. ClinicalTrials.gov

This proposal includes a trial that requires registration at ClinicalTrials.gov, in accordance with Public Law 110-85. The principal investigator is responsible for conducting the trial, has access to and control over the data from the clinical trial, has the right to publish the results of the trial, and has the ability to meet all of the requirements for submitting information under the law. Thus, the PI will register the proposal once the Choctaw Nation and OUHSC IRB have approved the study, but before any participants are recruited.

INCLUSION OF WOMEN AND MINORITIES

Inclusion of Women: It is expected that approximately half of the participants in the proposed studies will be female. Female participants of childbearing potential will be included in the study. Pregnancy testing will be performed as part of the screening visit, if determined to be appropriate by the health care provider. Female participants who are currently pregnant or plan to be become pregnant before the end of their potential involvement in the study will be excluded.

Inclusion of Minorities: The studies in this proposal will be open only to American Indian youth living within the Choctaw Nation Services area in Southeast Oklahoma. The population distribution for the state and region is shown in the table below.

Population Distribution for the state of Oklahoma and the 10½ county area of Southeast Oklahoma comprising the Choctaw Nation Service Area

	American Indian or Alaskan Native	White, not of Hispanic Origin	Black, not of Hispanic Origin	Hispanic or Latino	Asian or Pacific Islander	More than one race
Oklahoma (state)	8.0%	70.8%	8.1%	8.2%	1.7%	4.1%
SE Oklahoma (Choctaw Nation)	13.4%	73.0%	4.0%	4.4%	0.4%	5.4%

Source: US Census Bureau, Washington, DC, Last revised 04-Nov-2010, available online at: http://quickfacts.census.gov/qfd/states/40/40109.html

Human Research Participant Protection The University of Oklahoma IRB APPROVED AAHRPP
IRB NUMBER: 0434
IRB APPROVAL DATE: 01/04/2016

INCLUSION OF CHILDREN

a. **Inclusion of children:** All participants studied in this proposal will be children according to NIH definition.

b. **Rationale for specific age of children**: Males and females 11-21 years old will be enrolled. This age group of participants was selected because of the rapid increase in the prevalence of overweight/obese children and young adults in the past three decades and the likelihood that overweight/obese children will become obese adults, thus increasing the lifetime risk for developing metabolic and vascular disease. Additionally, the Choctaw Nation Health Care Center patient care records demonstrate that members of their community have higher prevalence of obesity, diabetes and related cardiometabolic disease. Our belief is intervening during youth with exercise and lifestyle modification approaches is critical to minimize current and future disease risk. Average spontaneous daily physical activity levels decline throughout childhood and adolescence so by the early-to-mid teenage years less than half of this population reaches the recommended 60 minutes per day of moderate-to-vigorous activity on 5 or more days per week. Thus, targeting an exercise intervention during adolescence is likely to have a clear impact on health and function outcomes.

c. **Expertise of the investigative team for dealing with children at the ages included:** The principal investigator and co-investigator, and named personnel of the study team have primary appointments in the Section of Diabetes and Endocrinology in the Department of Pediatrics at the University of Oklahoma Health Sciences Center. Dr. Kenneth Copeland is Chief of the section of Diabetes/Endocrinology since 1999. His expertise in research on the problems of obesity and diabetes in youth is exemplified by his role as principal investigator for Oklahoma and national Vice-Chair for the TODAY Study (NIDDK-supported multi-center trial of type 2 diabetes in youth). Dr. Kevin Short joined the section in 2006 and helped established the Pediatric Metabolic Research Program for performing the type of studies planned in this proposal. The broad goals of the section research team address the current obesity and diabetes epidemic in children and the role of exercise and lifestyle, the early influence of the in uteri and perinatal period, and the disproportionate burden of metabolic disorders in Native Americans.

Dr. Short's recent ongoing studies over the past 8 years examine the effects of either single exercise sessions or repeated exercise training performed by adolescent children on the same metabolic (e.g., fitness, body composition, and glucose tolerance tests) outcomes as proposed in this study. Thus, all procedures have been developed and successfully performed with children and adolescents and we view extended exercise training studies as a logical outgrowth of the current work.

In summary, the investigative team is well positioned with facilities and local professional expertise to perform the proposed research in adolescent children.

d. **Suitability of the available facilities to accommodate children:** The Choctaw Nation Health Care Center is a JCAHO-certified medical center with the requisite resources and staff to provide medical care to children and adults. Dr. Copeland regularly consults with pediatric patients with type 1 or type 2 diabetes, obesity and other endocrine disorders at this facility. The Diabetes Wellness Centers in Talihina and the additional Wellness Centers in Durant and Hugo have been designed to accommodate all members of the community, from children to elderly people. Each of the Wellness Centers currently operates after-school and weekend activities for children. In Oklahoma City our Metabolic Research Program is located adjacent to the Pediatric Diabetes/Endocrinology Clinics and staff offices on the fourth floor of the University of Oklahoma Children's Physicians Building. It includes laboratories and equipment for exercise testing and training, body composition, vascular function tests, and biochemical/molecular analysis of biologic samples (e.g., plasma/serum tests in this proposal) that have been designed for research on children and adults. Clinic and multi-purpose rooms are available for meetings with the participants and their families, performing exams, and phlebotomy. All of these resources in Southeast Oklahoma and Oklahoma City

Human Research Participant Protection The University of Oklahoma IRB APPROVED AAHRPP
IRB NUMBER: 0434
IRB APPROVAL DATE: 01/04/2016

are currently used to conduct investigations performed with children of the same age and similar background characteristics, including the TODAY study.

e. Inclusion of a sufficient number of children to contribute to a meaningful analysis relative to the purpose of the study: The number of children required to reach statistically and clinically meaningful outcomes was estimated from published data acquired from prior studies both in adults and children. Continuous evaluation and refinement of methods and outcomes will be performed so current and future studies can be rationally designed with the highest scientific value and participant safety in mind, while managing the number of children involved in the research.

Vertebrate Animals

Not Applicable

Select Agent Research

Not Applicable

Consortium/Contractual Arrangements

Not Applicable

Resource Sharing Plan

We will comply with NIH requirements to provide updates on project progress at the requested intervals. The study design and intent to complete the research will be made available through the registry at ClinicalTrials.gov in accordance with Public Law 110-85. We intend to share the results of the study through public dissemination at scientific conferences and through publication in peer-reviewed journals. We do expect new methods, reagents, research tools or other new products to arise directly from the conduct of the proposed project. However, there will be biological material (plasma/serum) and data sets that are generated that could have value to outside groups interested in collaboration. We will make such materials available to other researchers as long as the scientific merit, sample integrity and rights of the study participants are certain to be protected. NIH support of the project will be cited for all publications and presentations.

Letters of Support

Starts next page

Human Research Participant Protection
The University of Oklahoma
IRB APPROVED
AAHRPP
IRB NUMBER: 0434
IRB APPROVAL DATE: 01/04/2016
