# Supplementary material for: Using financial incentives to promote physical activity in American Indian adolescents: A randomized controlled trial
Source: PLoS One. 2018 Jun 1;13(6):e0198390. doi: 10.1371/journal.pone.0198390 (PMC5983431; doi:10.1371/journal.pone.0198390)
Supplement: S1 Table — Participants in Phase 3 (Weeks 33–48) were randomly assigned to either the Ramp-down or Raffle group. The Ramp-down group earned progressively less money each week. The bonus was provided to participants who completed three sessions in the corresponding week. For the Raffle group, the number of chances increased with both frequency and duration of exercise. Exercise sessions with less than 20 minutes of moderate-to-vigorous physical activity did not earn a payment or raffle chance, respectively. (PDF) [file pone.0198390.s003.pdf]

S1 Table. Payments for Phase 3

A. Ramp-down group payments:

	Time per session				
Week	20 min	40 min	60 min	Bonus	Weekly Total
1	$2	$4	$6	$2	$20
2	$2	$4	$6	$2	$20
3	$2	$4	$5	$2	$17
4	$2	$4	$4	$2	$14
5	$1	$2	$4	$2	$14
6	$1	$2	$3	$1	$10
7	$1	$1	$3	$1	$10
8	$1	$1	$2	$1	$7

B. Raffle group chances earned each week:

	Session of the week			
Exercise time per session	Day 1	Day 2	Day 3	Weekly Total
20 min	1	2	3	6
40 min	2	3	5	10
60 min	4	5	7	16
