# Supplementary figures and images for: Using financial incentives to promote physical activity in American Indian adolescents: A randomized controlled trial
Source: PLoS One. 2018 Jun 1;13(6):e0198390. doi: 10.1371/journal.pone.0198390 (PMC5983431; doi:10.1371/journal.pone.0198390)

**S1 Fig. Flow chart for Phase 3**

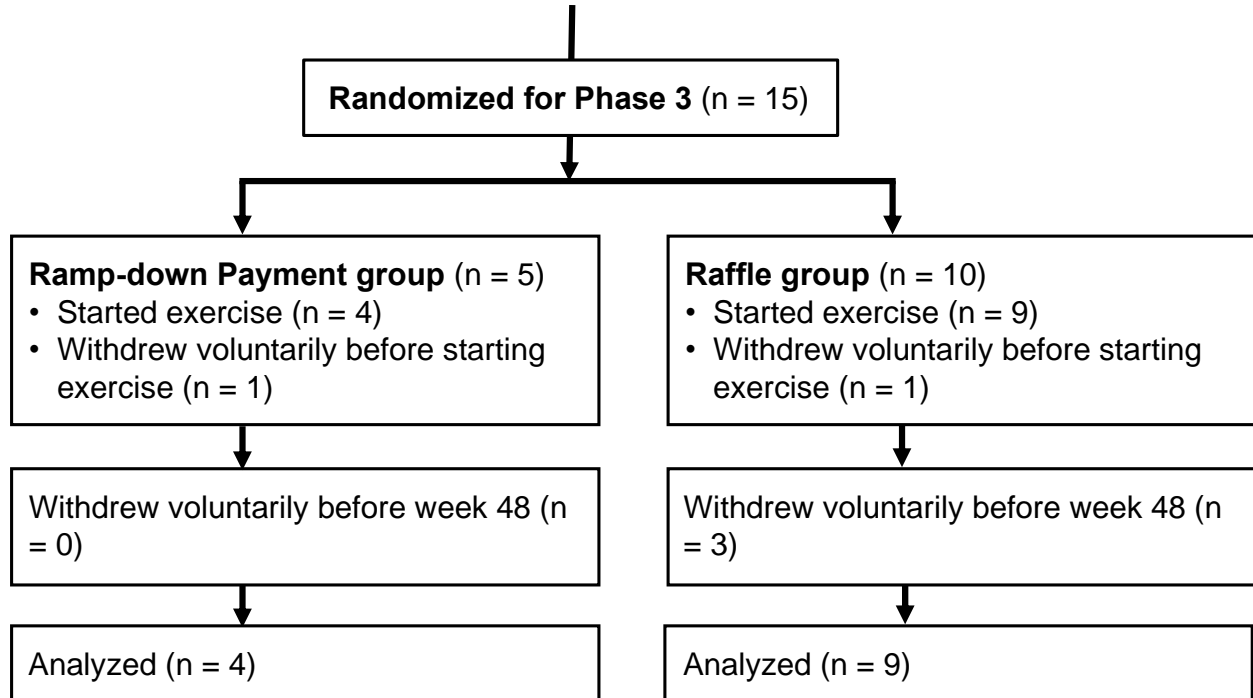

Supplement: S1 Fig — The Ramp-down group received diminishing payments while the Raffle group received discontinuous payments, as described in the text. (PDF) [file pone.0198390.s004.pdf]
